# Spatial cell type composition in normal and Alzheimers human brains is revealed using integrated mouse and human single cell RNA sequencing

**DOI:** 10.1038/s41598-020-74917-w

**Published:** 2020-10-22

**Authors:** Travis S. Johnson, Shunian Xiang, Bryan R. Helm, Zachary B. Abrams, Peter Neidecker, Raghu Machiraju, Yan Zhang, Kun Huang, Jie Zhang

**Affiliations:** 1 Department of Biomedical Informatics, The Ohio State University, Lincoln Tower 250, 1800 Cannon Dr., Columbus, OH 43210 USA; 2 Department of Medicine, Indiana University School of Medicine, Emerson Hall 305, 545 Barnhill Dr., Indianapolis, IN 46202 USA; 3 Department of Biostatistics, Indiana University School of Medicine, HITS 3000, 410 W. 10th St., Indianapolis, IN 46202 USA; 4 Department of Mathematics, The Ohio State University, Math Tower 100, 231 West 18th Ave., Columbus, OH 43210 USA; 5 Department of Computer Science and Engineering, The Ohio State University, Dreese Laboratories 779, 2015 Neil Ave., Columbus, OH 43210 USA; 6 Regenstrief Institute, 335, 1101 W. 10th St., Indianapolis, IN 46202 USA; 7 Medical and Molecular Genetics, Indiana University Purdue University Indianapolis, HITS 5015, 410 W. 10th St., Indianapolis, IN 46202 USA

**Keywords:** Cognitive ageing, Neural ageing, Cellular neuroscience, Next-generation sequencing, RNA sequencing, Computational neuroscience, Data integration, Microarrays

## Abstract

Single-cell RNA sequencing (scRNA-seq) resolves heterogenous cell populations in tissues and helps to reveal single-cell level function and dynamics. In neuroscience, the rarity of brain tissue is the bottleneck for such study. Evidence shows that, mouse and human share similar cell type gene markers. We hypothesized that the scRNA-seq data of mouse brain tissue can be used to complete human data to infer cell type composition in human samples. Here, we supplement cell type information of human scRNA-seq data, with mouse. The resulted data were used to infer the spatial cellular composition of 3702 human brain samples from Allen Human Brain Atlas. We then mapped the cell types back to corresponding brain regions. Most cell types were localized to the correct regions. We also compare the mapping results to those derived from neuronal nuclei locations. They were consistent after accounting for changes in neural connectivity between regions. Furthermore, we applied this approach on Alzheimer’s brain data and successfully captured cell pattern changes in AD brains. We believe this integrative approach can solve the sample rarity issue in the neuroscience.

## Introduction

An important goal for neuroscience is to understand how structural, anatomic, and cellular heterogeneity contribute to brain development, health, disease, and degeneration^1^. Brains have spatially-explicit functionality that contribute to cellular heterogeneity within each specific regions^2^. Cell types in the brain are incredibly diverse with vastly different functions, size, shape, and molecular profile that directly affect the function of anatomic locations^3^. Thus, there is a strong need to combine anatomically explicit tissue sampling with functional assessment to localize molecularly defined subtypes in brain tissues, while detecting morphology, activity, or connectivity at the same time^4^. To address this need, the Allen Human Brain Atlas (AHBA) previously curated spatially-sampled, tissue level transcriptomic data (microarray) from six human whole brain donors^5^, but without specific cell type and subtype information. It provides an excellent platform to carry out cell types deconvolution and study cellular heterogeneity across multiple human brains with their explicit spatial and anatomical information. However, the AHBA contains data from only six donors, but high-dimensional informatics analyses for deconvolution are best suited for large sample sizes.

The scRNA-seq technique has empowered a new generation of transcriptomic analysis that classify and characterize gene expression at the level of individual cells. It has enabled classification of individual cells based on gene expression profiles and led to identification of new, functionally-distinct subtypes of cells^6–9^. For example, pathways are differentially activated between single cells in the developing human cortex^10^ and microglia cells have altered transcriptional states during Alzheimer’s disease progression^11^. More importantly, this approach identifies cellular processes and pathways that are differently regulated among cell types, conditions, and location in tissue. Cell type composition plays a very important role neurological morphology, development, and degeneration. Therefore, it is of the upmost importance to apply this technique for spatial brain cell type mapping. Recently human scRNA-seq has been applied to estimate the cellular proportions from tissue samples in multiple tissue types^12–14^. However, scRNA-seq data generally has very large variance among studies, which may be due to factors such as the experimental platform, library preparation process, and species^15^.

For this reason, we explore the possibility of completing so-far limited human brain RNA-seq datasets from both bulk tissue studies such as AHBA and scRNA-seq studies with relevant model animal data, combined with neural connectivity information for better brain cell type spatial deconvolution in human samples.

This approach poses two important assumptions: First, we assume that evolutionary homology between the model organism and human captures similar biological system dynamics; second, we assume that integration of these model organisms improves what can be achieved by examining cellular heterogeneity from human scRNA-seq datasets alone. Obviously, the brains of model animals differ from human ones and biological inferences cannot be expected to transfer without caution. For instance, the human brain structure has undergone evolutionary expansion in comparison to mouse brain, most notably in the distinct division between human L2 and L3 cortex in contrast to mouse^16^. Furthermore, some molecular and functional characteristics of brain cells have changed between mouse and human^17^. A notable example is ubiquitous HCN1-subunit expression in human neurons and associated h-channel membrane properties absent in mouse neurons^18^. However, conserved anatomical structures and cellular functions are still observed in the mouse and human brains. For example, the gross anatomy^16^ and broad cell type classes^17,19^ are consistent between human and mouse. Certain layer specific cortical pyramidal neuron subtypes show consistency between human and mouse^14,20^, interneurons share similar gene co-expression signatures^21^, and region-specific gene expression is homologous between mouse and human^22^. Furthermore, feature reduction^14^ or feature selection^19^ through classical statistics and machine learning can correct for some interspecies variance in brain tissues. All of these studies let us hypothesize that an integrative approach to identify transcriptomic features from mouse can contribute to the completion of the cell type features absent in the human brain scRNA-seq data and better deconvolute spatial cell composition in the whole brain level.

In this study, we used two separate human brain scRNA-seq datasets, each completed with cell type features obtained from mouse scRNA-seq data. We then predict cell type compositions and create spatial maps of cell types for microarray data from all six AHBA human brains. We then compared the prediction results among features from the mouse data only, and from the two separated human datasets each completed with mouse features.

We perform integrative transcriptomic feature selection with the mouse and human scRNA-seq data, complete absent cell features from human scRNA-seq with corresponding cell type features from mouse scRNA-seq data, then applied non-negative linear models to estimate cell type proportions for nine major cell types/subtypes in the six AHBA brains (interneuron, S1 pyramidal, CA1 pyramidal, oligodendrocytes, microglia, astrocytes, endothelial, ependymal, and mural cells). We observed spatially consistent estimated cell type proportions in all six normal AHBA human brains when using mouse data alone, as well as using the combinatory features from both species as input.

Aside from the general consistency in normal adult human brains, we also observed consistent cell type deconvolution results with previously established neuron and microglia abundances in Alzheimer’s disease (AD) brains^23^. The neuron to glia ratios were correlated with previously established nuclei counts after accounting for changes in neural connectivity in different brain regions. Moreover, the ratios derived from RNA were significantly correlated with clinicopathological measurements for AD progression.

Single dataset, especially scRNA-seq datasets, are prone to bias through platform, species, and high dropouts^14,15,24^. Signals identified across multiple datasets with respect to a phenotype are usually considered with higher confidence. Similar to meta-analysis, combined scRNA-seq results are usually more robust than any single study/dataset. In this case, it is critical to determine consistent signals, i.e. genes across dataset, species, and platform. Indeed, there has been some success using feature selection to integrate scRNA-seq data in other tissues for cell classification^25^.

There have even been attempts at complete deconvolution where no single cell or bulk expression profiles are known for cell types. CDSeq is one such example, which relies on matrix factorization to identify both the cell type signals and the cell type proportions simultaneously^26^. Alternatively, mutual linearity of genes has also been used for complete deconvolution^27^. In this study we focus on the case where scRNA-seq information is available and therefore can be used for deconvolution. It has been shown that multiple scRNA-seq datasets can be used simultaneously for deconvolution^14^ and that linear models still outperform most deconvolution algorithms^28^. Wang et al. estimated cell type proportions in bulk RNA-seq using scRNA-seq from multiple human pancreases using the MuSiC algorithm but did not integrate microarray to scRNA-seq or mouse data to human^14^. Similarly, the SCDC algorithm deconvolutes bulk sequencing using scRNA-seq from multiple source by training an ensemble model derived from each source^29^. Tsoucas et al. found that weighted linear models performed the best in deconvoluting tissue samples with scRNA-seq but did not use multiple datasets to reduce bias^28^. We therefore chose linear models to deconvolute brain samples. This is the first study to perform integrative feature selection on cell types involving different organism to fill in missing information in human brain. For these reasons, we used feature selection to identify gene sets that are consistently expressed across species but not across cell types. Thus, these genes differentiate cell types within and among samples and even across species.

Because brain neurons usually have large size and irregular shape, spatial location using the nuclei alone are usually not adequate cell type identification^3^. People have found that neural connectivity is a covariate of neuron size and axon projection length and can be used to localize neurons^30^. We investigate the association between neural connectivity of different brain regions and cell type distributions inferred from our study.

Previous applications of scRNA-seq deconvolution assume that expression levels are unaffected by general differences in RNA quantities among various cell types, but differences in RNA quantity may alter differentiation among cell types. For example, neuronal cells show size variation among brain regions, and neurons are consistently larger than glia. A recent study addressed this problem by incorporating a cell size correction into the DESCENT tool^13^. All of these technical considerations will play a large role as deconvolution algorithms are increasingly used to explain clinical outcomes in neurology.

Our contribution to this line of work is the use of model organisms to “fill in” missing cell types during deconvolution experiments, which is a big challenge and key problem to solve in the human brain research where samples are usually scarce and very difficult to obtain. We are able to achieve this goal using our iterative feature selection approach and linear model methods for deconvolution. Because linear models have been well established for deconvolution, we applied that so that we can focus more on resolving the spatial changes of cell type composition through the brain and to directly study the affects of neural projections on deconvolution results.

## Results

### Model validation: estimation of cell types in simulated scRNA-Seq data

**Figure 1:**
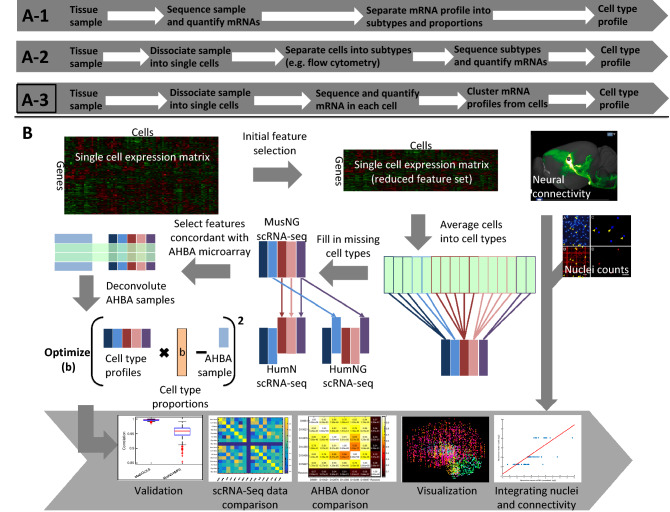
The classic characterization of cell types from RNA-Seq data. (**A-1**) and (**A-2**) are conventional workflows for cell type specific expression acquisition before the advent of scRNA-Seq. (**A-3**) is the current state of the art workflow, and was chosen as the basis for cell type characterization in this study. (**B**) The workflow in this study. *MusNG* mouse neuron and glia cell scRNA-seq dataset, *HumN* human neuronal cell scRNA-seq dataset, *HumNG* human neuronal and glia cell scRNA-seq dataset.

For this study, we used three scRNA-seq datasets, one from mouse brain (denoted as MusNG^8^), two separate datasets from human (denoted as HumNG^6^ and HumN^7^ respectively, Supplementary Fig. 1). Before applying the workflow shown in Fig. 1 to deconvolute AHBA microarray data, in order to test and validate our feature selection protocol and find the best linear modeling approach, we first examined how well the models predicted cell types from simulated data, i.e. aggregated scRNA-seq data. Specifically, the simulated data was generated by randomly sampling 50% of the cells from the MusNG, HumN + MusNG, or HumNG + MusNG dataset and averaging across the cells for each gene. The other 50% of cells were used to generate the cell type expression profiles for feature selection. We compared two different classical statistical models and one published deconvolution method on the correlation of estimated cell type compositions to the ground truth cell type compositions. The two classical statistical models we used are ordinary least squares regression (OLS) and non-negative factorization regression (NMFR). The published single cell deconvolution method we compared with is Dampened Weighted Least Square (DWLS)^28^.

**Figure 2:**
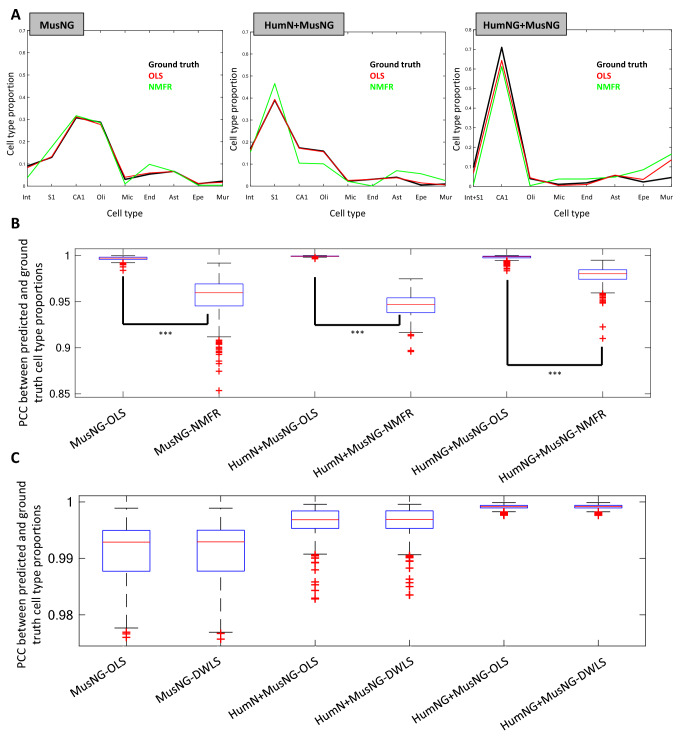
A comparison of cell-type estimate PCC using the data from each of the three scRNA-Seq datasets. (**A**) Representative examples of the cell-type proportions for each scRNA-Seq input on Donor 10021. *MusNG* A MusNG simulated sample deconvoluted using the cell types from MusNG, *HumN + MusNG* A HumN + MusNG simulated sample deconvoluted using the cell types from HumN + MusNG, *HumNG + MusNG* A HumNG + MusNG simulated sample deconvoluted using the cell types from HumNG + MusNG. (**B**) Pearson’s correlation coefficients (PCC) distribution between the true proportions of cell types from simulated tissue samples and predicted proportions across nine cell types among repeats for the OLS and NMFR comparison. (**C**) PCC distribution between true proportions of cell types from simulated tissue samples and predicted proportion baseline comparison between DWLS and OLS using the same gene set. For (**B**) and (**C**) the experiments were repeated 100 times for each scRNA-Seq dataset, regression method, and Allen Human Brain Atlas (AHBA) donor. ***Indicates p-value < 0.001.

The predicted proportions of cell types were consistent with the known proportions of cells in the simulated data (Fig. 2A) for both methods in all three simulated datasets. However, we found that OLS performed better than NMFR with higher accuracies for all cell types (Fig. 2A). Moreover, there were much higher and very consistent Pearson correlation coefficients (PCC) values across nine cell types among repeats by OLS than by NMFR (Fig. 2B). To further demonstrate that OLS is an effective approach, we also compared it against another commonly used deconvolution technique DWLS. We found that the PCC among the same 9 cell types for DWLS and OLS were comparable (Fig. 2C) using the same feature set. Because OLS is simpler to implement, interpret, requires less computation, and performed comparably in our comparison, we chose OLS for all further analyses.

### Cell types are spatially consistent in normal human brains

#### All scRNA-seq datasets generate consistent results

**Figure 3:**
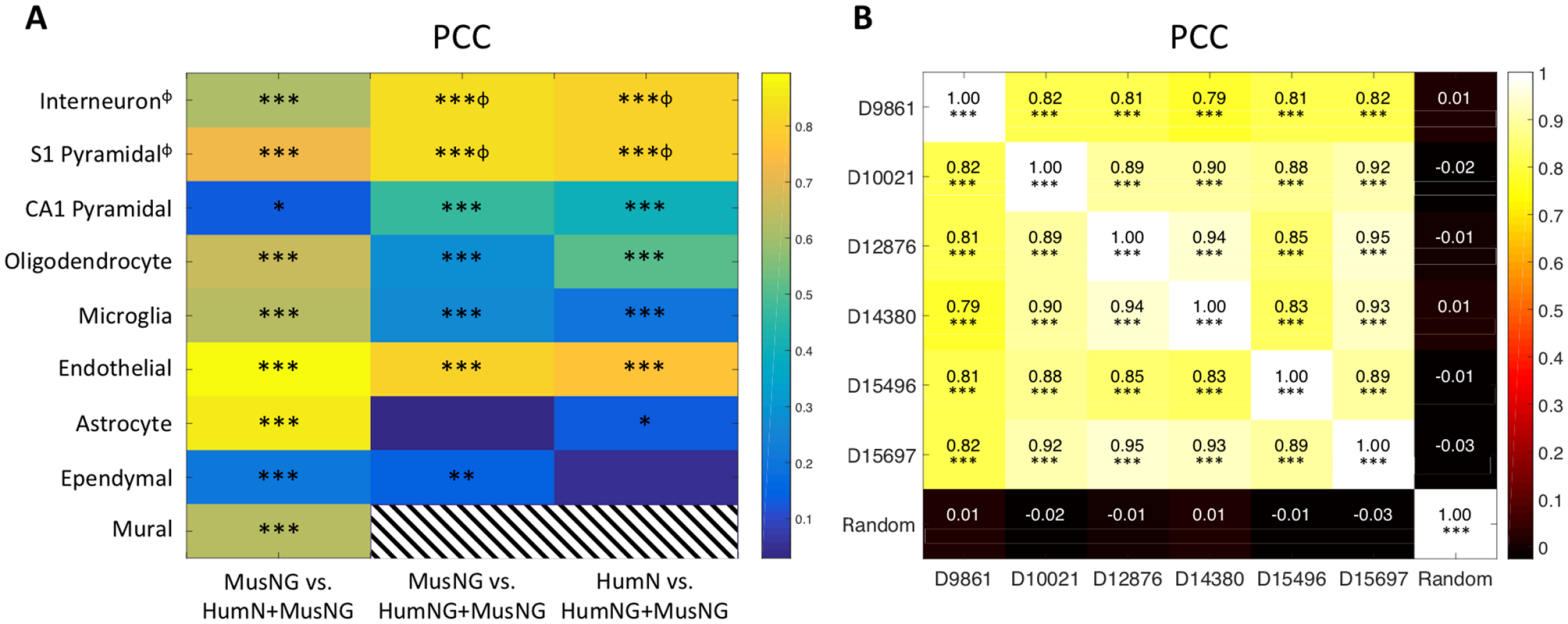
Pearson’s correlation coefficients (PCC) for each cell-type and donor (**A**) Mean PCC values for each cell-type correlation across all samples from the six donor brains, each deconvoluted with different input scRNA-Seq datasets. See Supplementary Fig. 1 for more information. Stripes indicate that there were not enough data to generate a correlation. (**B**) Donor-to-donor consistency for cellular compositions prediction from the three input scRNA-seq datasets. Mean Pearson’s correlation coefficient (PCC) over all three results and over all anatomic locations. *Indicates correlations p < 0.05, **p < 0.01, ***p < 0.001. ^ϕ^Indicates where cortical neuron was used instead of interneuron and S1 pyramidal cell, i.e. from HumN and HumNG.

To see whether the input scRNA-seq data affected the results of deconvolution, we compared the deconvolution results on the microarray data from six AHBA donors with every pair of input scRNA-seq datasets (i.e. MusNG vs. HumN + MusNG, MusNG vs. HumNG + MusNG, HumN + MusNG vs. HumNG + MusNG). The cell-type proportions were estimated in each of the six AHBA donors using each input scRNA-Seq dataset (i.e., MusNG, HumN + MusNG, or HumNG + MusNG; see Supplementary Fig. 1 and “Materials and methods” for details). For each donor brain, all microarrays were used, which ranged from 363 to 946 samples. The mean (across all AHBA donors) cell proportions for each cell type were compared. We tested whether the spatial cell type proportions are spatially consistent (indicated by PCC values across six donors) with respect to input scRNA-seq data. The resulting estimated cell-type proportions were consistent regardless of the scRNA-Seq dataset, as indicated by high and significant PCCs among majority of the cell types (Fig. 3A).

For individual cell types, we expected cell-type correlations among different input datasets to be positive, which would suggest that the cell-type proportions are consistent between input datasets. We observed that 91% (21/23) of the possible correlations were significantly positively correlated (Fig. 3A) with significant P values (P < 0.05). Neural cell-type correlations were stronger than glial cell-type correlations—possibly because glial cell types were harder to detect (Supplementary Figs. S4–10). As expected, the correlations between the same cell-type were more frequently positively correlated than the ones for mismatched cell-type among different input datasets (Supplementary Figs. S4–10, Supplementary Table 2). The results show that regardless of the input scRNA-Seq datasets, the spatial distribution of cell types were generally consistent in normal human brains, which demonstrated the feasibility and effectiveness of our feature selection and deconvolution workflow.

Furthermore, the cell-type estimates using the MusNG dataset were more similar to the HumN + MusNG datasets for AHBA donors than to the HumNG + MusNG. The cell type estimate correlations between input HumN + MusNG and HumNG + MusNG were higher than the MusNG vs. HumNG + MusNG correlations (Fig. 3A, Supplementary Figs. S4–10). We believe this is due to the higher gene expression profile similarity between HumN and HumNG datasets. Interestingly, the results also indicated that some dissimilar cell types follow similar spatial patterns both within and between datasets. For example, human oligodendrocyte proportions correlate with mouse astrocyte proportions (Supplementary Figs. S4–10B,D,F). This finding could be attributed to similar glial expression profiles or an overlap between the two cell types in anatomical space. We also observed that the absolute proportions of neural cell types, especially pyramidal cells, were much higher than glial or interneuron cell types (Supplementary Fig. S4–10A,C,E) in cerebrum samples. This finding may be attributed to the higher quantity of neural cell mRNA or the characteristic mRNA profile for neural cell being easier to detect.

#### All AHBA donors produce a consistent spatial cell type map

In this section, we checked if the individual differences from brain donors affect the cell-type proportions by comparing the spatial cell type composition for every pair of the six donors.

For each anatomic location, the mean cell type proportions were calculated across multiple samples and across three input datasets (MusNG, HumNG + MusNG, HumN + MusNG). For example, if 10 estimated proportions for each of the nine cell types were obtained for a specific anatomic structure using each of the three inputs, then the mean was taken across all 10 proportions for each cell type, resulting in a single vector of nine cell-type composition values. These aggregated cellular compositions were generated for each anatomic location as a distribution of cell types and could be compared across donors. We observed high cell-type consistency for all anatomic locations: the average pairwise PCC was 0.87 ± 0.07 across all three scRNA-Seq datasets (Fig. 3B). The PCCs among AHBA donors were also significantly higher and uniformly positive than randomly shuffled data (Fig. 3B) with none of the latter significantly correlated to any real donor (Fig. 3B, Supplementary Fig. S11–13). Despite the general consistency and as one might expect, we also observed some variability among brain donors. For example, AHBA donor D9861 had significantly lower correlations than other donors (p-value = 1.46 × 10^–7^ by t-test), which indicates that D9861 may have some neurological or physiological condition that is different from the other donors. Aside from inter-donor consistency across all cell types, we were also interested in the consistency of results for individual cell types across species.

Overall, the results show that individual brain differences do not affect the cell type spatial deconvolution significantly, and we were able to generate a very consistent spatial cell type map using either mouse scRNA-seq data or human scRNA-seq data supplemented by mouse data.

### Major cell types previously known to be enriched in certain areas are spatially localized in expected regions.

**Figure 4:**
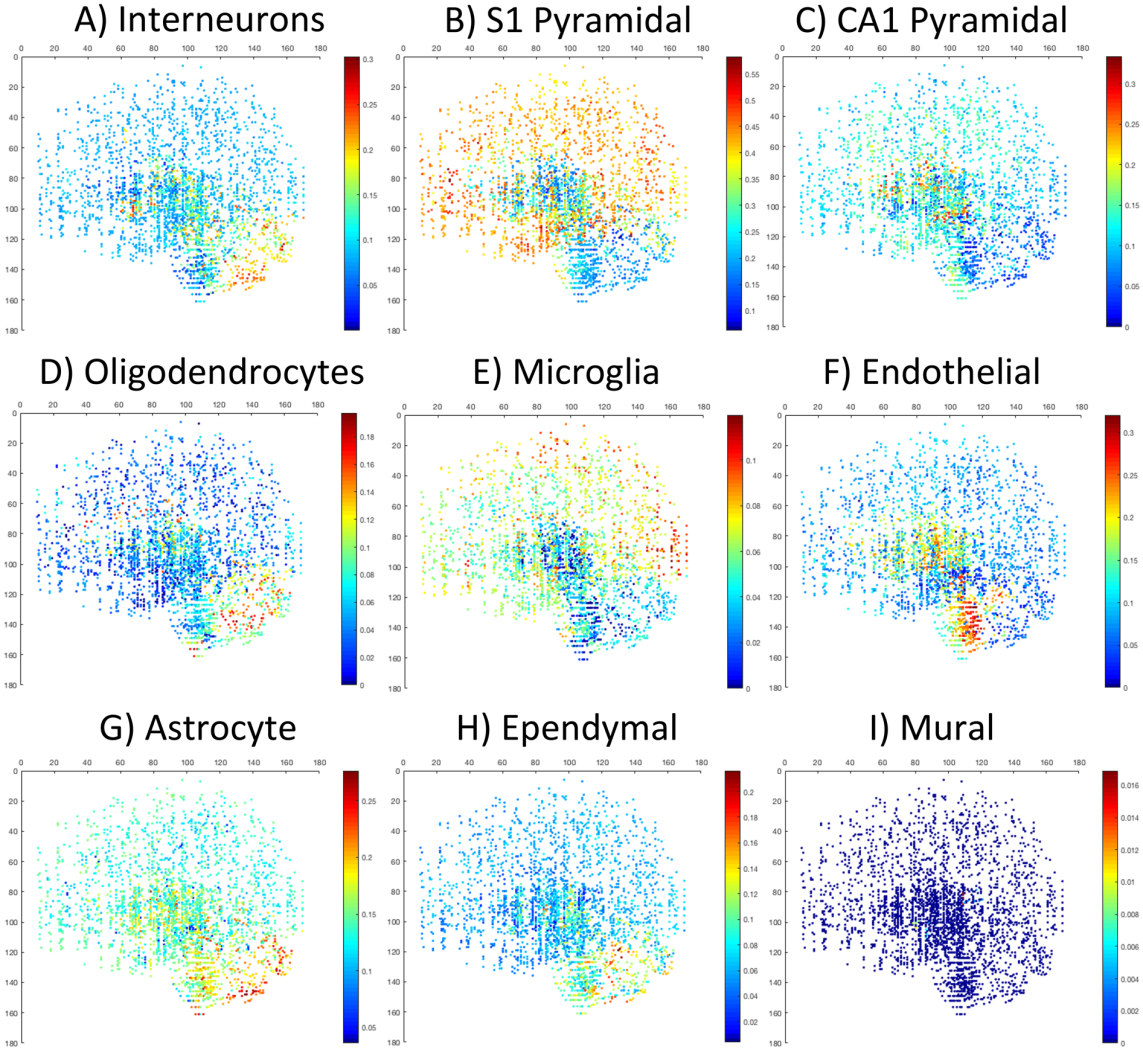
The proportion of each cell type plotted from a sagittal view. The proportions were measured for the following MusNG cell types: (**A**) Interneuron, (**B**) S1 pyramidal, (**C**) CA1 pyramidal, (**D**) oligodendrocyte, (**E**) microglia, (**F**) endothelial, (**G**) astrocyte, (**H**) ependymal, and (**I**) mural. See individual scale bars for proportions.

With the previous knowledge of certain cell types mostly enriched in certain brain regions^31^, we also examined if those cell types were mapped to the expected anatomic locations and if the input data from different species matters. We found that regardless of using mouse input data or mouse supplemented human data, those cell types were correctly mapped to the expected location in the AHBA brains. For example, the S1 pyramidal cell inferred by MusNG corresponding cell features were localized to the human cortex and the CA1 hippocampal cell type inferred by MusNG were localized to the human hippocampus region in the AHBA donors (Fig. 4B,C, Supplementary Figs. S14–31C2–3). Endothelial cell types were also localized to human brainstem regions regardless of input data species, where many large blood vessels enter the brain (Fig. 4F, Supplementary Fig. S14–31C6). Ependymal cells were localized to human ventricular regions of the brain (Fig. 4H, Supplementary Fig. S14–31C8). These results show that general cell type expression profiles are consistent across mouse and human, and the cell type composition vary significantly among brain regions, as previously known^23^.

**Figure 5:**
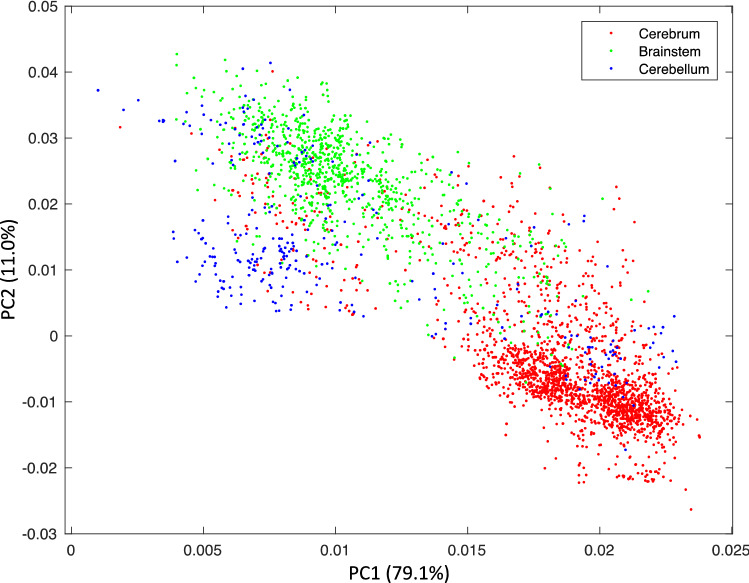
Clustering of cell-type proportions in all AHBA samples and overlay with three major brain regions. Principal component analysis (PCA) plot of the cell-type proportion matrix resulted from all 3702 microarray samples from all AHBA brains, averaged over three different scRNA-seq inputs. The colors indicate the three regions (cerebrum, brainstem, and cerebellum) of the sample.

Since cell type proportions are more homogeneous within a brain region than across regions, we further checked if certain cell types were correctly mapped to a major brain region in general. To do this, we first performed principal component analysis (PCA) on all cellular composition estimates from every microarray sample of the six donor brains. Then we performed K-means clustering on those values for all donor samples (Fig. 5). We discovered that, regardless of input scRNA-seq dataset and AHBA donor, the data points (PCA components of cell proportion estimates from each microarray samples) are well separated into three clusters with few overlaps. Not surprisingly, these clusters corresponded well to the known anatomic regions from which the sample was taken in the donors brain, namely cerebrum, cerebellum, and brainstem (Fig. 5). The mean accuracy between the clusters and the true brain regions for all combinations of AHBA donors and scRNA-Seq datasets was 81 ± 7% (Supplementary Table 1). The sensitivity and specificity of these clusters localized to the true brain region were also analyzed with respect to the donors, scRNA-Seq datasets, and brain regions (Supplementary Table 2). We found high average sensitivity (0.80 ± 0.16) and high specificity (0.91 ± 0.05) across all scRNA-Seq datasets, brain donors, and brain regions (Supplementary Table 2). The high sensitivity and specificity demonstrate that we can confidently conclude that the cell-type proportions identified from our workflow mapped very accurately to the major brain region from which each sample is extracted.

We also discovered that with the workflow and model established in this study, anatomic region was the only major cofounding factor for the sensitivity and specificity of the anatomic region clustering. We analyzed the major cofounding factors for the cellular composition clustering with multivariate analysis of variance (MANOVA). The p-values for the sensitivity and specificity with scRNA-Seq dataset, AHBA donor, and brain region were 0.3083, 0.2601, and 0.0019, respectively (Supplementary Table 2, Fig. 5). These results showed that only brain region significantly affects the ability to cluster the cell-type composition into the correct anatomical regions. For example, it was more likely for brainstem samples to have cell-type compositions similar to the cerebellum or cerebrum than for cerebrum or cerebellum samples to have non-region-specific cell-type composition (Fig. 4, Supplementary Figs. S14–31B, Supplementary Table 2). More importantly, deconvolution results were consistent for different scRNA-Seq input datasets and AHBA donors. This finding further validates our feature selection and deconvolution techniques when mouse scRNA-Seq data is used to fill in human brain expression data for cell-type location analysis.

### Neuron to non-neuron ratios are consistent with that from nuclei counts after correcting for projection size

**Figure 6:**
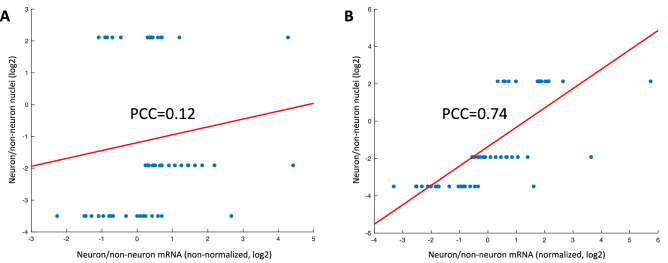
Consistency of nuclei proportion derived from staining-based nuclei counts with mRNA-based estimates, without and with adjustment for neural connectivity. (**A**) The neuron/non-neuron ratios between the nuclei-staining-based estimate and the unadjusted mRNA-based estimate. The 18 points on each tier of the y axis are the results from using the 3 RNA-Seq datasets on six Allen Human Brain Atlas (AHBA) donors. The three levels of nuclei ratios are for each region (cerebrum, cerebellum and brainstem). (**B**) The data are the same as in A except that the mRNA neuron/non-neuron ratios were divided by the projection volume, which adjusts for the differences in neuron length among different regions.

Moreover, we found that the neuron/non-neuron ratios derived from mRNA deconvolution did not initially match those derived from neuron nuclei counts^23^. The correlation between the nuclei-based ratios and our mRNA-based ratios across the cerebrum, brainstem, and cerebellum were not significant (Fig. 6A; PCC = 0.1282, p-value = 0.3556). We also noticed that the mRNA-based neuron/non-neuron ratios (p-value < 1.00 × 10^–16^ by ANOVA) and connectivity volume (p-value = 4.23 × 10^–15^ by ANOVA) varied significantly by brain region. However, after we adjusted for the neuron projection volume, i.e., dividing the mRNA-based neuron/non-neuron ratios in each region by the mean projection volume for that region, the PCC significantly improved (Fig. 6B; PCC = 0.7429, p-value = 1.26 × 10^–10^). We conclude that for neuron cell types, which have axons that travel long distances, the nuclei are not the optimal indicator for its contribution of total mRNA to the bulk tissue sample. Instead, neuron connectivity is a more effective indicator of the mRNA proportion for the neuron in the brain tissue.

### Neuron and microglia ratios correlate with AD clinical traits

As it is well established that the altered cellular composition in AD, characterized by neuron loss and microglia proliferation, is associated with AD progression and cognition status^32–34^, we then evaluated whether our method using mouse signatures identified via mouse scRNA-seq could detect cellular composition correctly in AD brains and if they match the AD progression or cognition status from the bulk RNA-seq data of AD and control samples. First, we used our deconvolution method to estimate the cell type proportions of Alzheimer’s Disease (AD) and normal brain samples from bulk RNA-seq data. Then, we tested the association between cell type proportions and AD clinical traits. The AD clinical traits we used include AD neuropathology represented by amyloid plaque scale (Plaque Mean) and cognitive status measured by Clinical Dementia Rating scale (CDR), Braak stage score (BBS). As a result, we observed a significant negative correlation between neurons (Interneuron, S1_Pyramidal, CA1_Pyramidal) and all the three AD clinical traits (Table 1). We also detected a significant correlation between microglia cells and all the three AD clinical traits (Table 1). The results verified that our workflow using mouse signatures could correctly predict cellular composition patterns of human AD brain samples consistent with AD pathology characteristics.

**Table 1:** Pearson correlation coefficients of neuron and microglia ratios with AD pathological scores.

	Interneuron	S1_Pyramidal	CA1_Pyramidal	Microglia
CDR	− 0.27***	− 0.24***	− 0.22***	0.17***
Amyloid Plaque mean	− 0.20***	− 0.18***	− 0.21***	0.19***
BBS	− 0.25***	− 0.17***	− 0.25***	0.18***

***Indicates p < 0.001.

## Discussion

Computational deconvolution is an important tool for transcriptomic data analysis, which not only elucidates cell-type specific transcriptomic profiles in future studies^12,13,26–29,35,41^, but also generates spatial cell maps which are very important to study regional functionality and disease-specific alteration in neuroscience. These approaches are generally limited to a single platform and usually for a single species^14,35^. Due to the limitations of sample preparing techniques and the rarity of human brain samples, the majority of the transcriptomic data are still from tissue mixture with multiple cell types. With the emergence of single-cell RNA-Seq technique, it becomes important to use single-cell gene expression to deconvolute and “purify” previously curated bulk tissue transcriptomic data to obtain representations of gene expression profiles for each cell type. In this study, we demonstrated that cell-type proportions could be derived with high accuracy through an inter-species deconvolution workflow. In fact, mouse brain expressions have already been used to study mental disorders^36^ such as autism spectrum disorder^37^, depression^38^ and schizophrenia^39^. Another study has shown that regional gene expression in mouse and human brain are concordant^22^. It has been shown that major neural cell types can be mapped between mouse and human if features are selected properly^19^. We demonstrated a high cellular composition consistency among a variety of input scRNA-Seq datasets using our workflow. We believe that integrative transfer-learning approaches to supplement human scRNA-seq with much more abundant and high-resolution model animal data can improve the deconvolution quality and greatly facilitate transcriptomic studies in neuroscience research.

We found in this study that in normal human brains, the spatial cell type patterns were highly consistent. AHBA brain donors all shared similar spatial patterns of cell types regardless of the scRNA-seq dataset used to obtain the cell type features. Furthermore, a simple linear model can accurately recapitulated true cell type proportions. Although more sophisticated non-linear methods can be used, we found that a simple linear model is easier to implement with adequate accuracy, and more importantly, was more interpretable.

A potential limitation of using linear models lies in the fact that it cannot capture nonlinear factors related to the cell abundance estimation. One example of such nonlinear factors would be cell–cell interactions that may result in the deconvolution to be non-linear. Furthermore, other methods like MuSiC and SCDC leverage sample information as a covariate for overlapping cell types when they train their models. We are considering the case where missing cell types are included from different species without including sample as a covariate for overlapping cell types. Additionally, we did not perform complete deconvolution as it is not necessary here, since our primary concern is the use of closely related species to complete human cell type expression profiles. However, if using complete deconvolution techniques, the cell type expression profiles and the proportions of those cell types could be determined simultaneously. With these potential limitations in mind, we chose to focus on using simple proven methods like OLS to study primarily on how speciation, neural connectivity, and spatial location affect deconvolution.

With this study, we discovered that when the cell type proportions were clustered into the principle cell types, they clearly aggregated to the corresponding spatial locations, namely the cortex, cerebellum, and brain stem, each with distinct cell compositions. Additionally, the mapping accuracy between cluster and brain region is not much different between mouse and human scRNA-seq completed with mouse data as input. Also, the individual donor difference does not significantly affect the results either. The only cofounding factor that may affect the cluster accuracy was the brain region itself, indicating that some brain regions, i.e. brain stem, were less distinct than others.

Furthermore, to apply our established workflow to real disease study, we tried our workflow on a large AD brain cohort study. It successfully predicted the neuron and microglia proportions, which are correctly correlated with AD pathological changes. We find that across three clinical tests of AD severity, three neuronal cell types, and one microglia cell type are associated with AD severity as expected. The AD samples were primarily taken from the cortex.

The previous study by Azevedo et al. generated the nuclei count data in specific brain regions by DAPI and NeuN protein staining^23^. Azevedo et al. used the counts of all nuclei (DAPI+) and neuron nuclei (NeuN+) to extrapolate the proportions of neurons in a specific anatomic region. However, the results did not initially match our neuron population estimate very well. The discrepancy between our unadjusted neuron to non-neuron ratios and those derived from the nuclei counts indicates that nuclei counts are not necessarily adequate for evaluating the amount of mRNA in neuron due to the large variation of size and shape of neurons among brain regions. Instead, using neural connectivity as a proxy for neuron size and mRNA quantity—i.e. bigger cells in general would have higher connectivity and also more mRNA. We adjusted the estimated neuron to non-neuron ratio of major brain regions from our deconvolution with the connectivity in that brain region. This correction generates highly correlated neuron/non-neuron ratios between our estimates and the nuclei counts.

In lieu of the general successes of or methods, we also identified caveats. The brain is an incredibly complex organ, which contains many cell types, and we only account for primary neuron cell types, which include hippocampal pyramidal cells and cortex pyramidal cells. More cell types and subtypes could be deconvoluted if corresponding scRNA-seq data from either mouse or human are available. The model itself can also be improved. For instance, brain cells, especially neurons, are constantly interacting with other cells in the brain. Since the signaling of these cells are through mRNAs, proteins, and metabolites, interactions between genes and cells should be accounted for, which may result in a more comprehensive model. Theoretically, this could be implemented using deep transfer-learning or interaction terms in the linear model.

## Conclusion

In this study, we estimated spatial cell-type composition across the entire AHBA brain transcriptomic data by deconvoluting each of the 3702 AHBA microarray samples with gene expression profiles of nine major cell types from both mouse and human scRNA-Seq. Highly consistent cell-type spatial mappings were achieved across all AHBA donors, which were also confirmed by known cell types in expected enriched regions. Furthermore, we found that the most conspicuous changes in cell types occurred between major anatomic regions, including the cerebrum, brainstem, and cerebellum. We also discovered consistent spatial cell-type relationships, such as mouse hippocampal pyramidal cells localizing to those in human hippocampus. We also showed that both nuclei location and mRNA location should be considered when localizing neural cells, due to their large irregular shape. Furthermore, we applied our workflow to AD brain data, and showed that using mouse neuron and microglia profiles, we can correctly estimate the increased microglia and reduced neuron population as AD progresses. In summary, we believe that when limited human scRNA-seq data is available, supplementing human scRNA-seq data with that from mouse or other model animal data can improve the cell deconvolution results for human brain bulk transcriptomic analysis, and greatly facilitate the functionality and disease study in neuroscience research.

## Materials and methods

### Dataset information

#### Mouse scRNA-seq dataset

The mouse scRNA-Seq dataset^8^ contained 3005 cells from mouse hippocampus and cortex, including both neural and glial cell types (denoted as MusNG, i.e., Mouse Neural-Glial). CD-1 mice and 5HT3EGFP transgenic CD-1 mice of both sexes between the 21–31 days postnatal were used in our experiments. The sequence reads were generated with the Fluidigm C1 protocol in the original study^8^.

#### Human scRNA-seq datasets

Two human scRNA-Seq datasets were used in this study. One dataset contained 3086 cells of neural cell types only from the cortex (denoted as HumN, i.e., Human Neural)^7^. This dataset was from living donors where tissue was resected for neurosurgical procedures. The nuclei libraries were prepared using Fluidigm C1. The second dataset contained 285 cells (both neural and glial cell types) from the temporal lobe^6^ (denoted as HumNG, i.e., Human Neural-Glial). The dataset was generated from an adult human brain tissue during epilepsy surgeries. The whole cell libraries were prepared using Fluidigm C1. Since MusNG is the most complete dataset in terms of the number of regions sampled and the diversity of cell types included, the MusNG dataset has been used to fill in missing cell-type expression data in the human datasets (Supplementary Fig. S1).

#### Normal human microarray data from AHBA

Six microarray datasets from the AHBA were analyzed by cell deconvolution in this study, one for each donor brain, each contains between 363 to 946 microarrays sampled throughout adult human brains^5^ with annotated region information and spatial coordinates. In total, 3702 microarrays were used. Each microarray sample represents tissue-level expression for its spatial location, facilitating the spatial mapping of gene expressions for the whole brain. Normalized microarray data were downloaded. A more detailed description of how the data was generated and normalized is available from the Allen Brain Institute (https://human.brain-map.org/).

#### AD human RNA-seq

Mount Sanai Brain Bank (MSBB) human Alzheimer’s Disease brain normalized RNA-seq samples was obtained from the Accelerating Medicines Partnership-Alzheimer’s Disease (AMP-AD) Knowledge Portal (synapse ID: syn20801188) with multiple brain regions (Frontal pole, Superior temporal gyrus, Parahippocampal gyrus, Inferior frontal gyrus). There are 975 samples in the MSBB cohort, which include normal, probable AD, possible AD and definite AD brain samples, the clinical (clinical dementia rating) and neuropathological (Braak stage score, Plaque Mean) traits separately.

#### Mouse neuron connectivity data

The brain connectivity dataset was generated from Drd1a-Cre_EY262 mice of both sexes at a wide variety of ages and contained all major anatomic regions (https://connectivity.brain-map.org)^3^. Specifically, recombinant adeno-associated virus (rAAV) and biotinylated dextran amines (BDA) tracers were used and the injection site volume and target site volumes were recorded for each tracer. These viral tracers were used to map the path of neural projections throughout the brain. The volume of a neuron projection shows how large the axonal projection of a neuron is. In our study, the sum of the projection volumes of injection and target sites was used as the measure of connectivity.

### Data preprocessing and feature selection

For both scRNA-seq and AHBA microarray datasets, z-score transformation of the expression was performed sample-wise so that each sample was individually converted to z-scores based on the mean and standard deviation of the expression values in that sample.

Mouse scRNA-Seq and AHBA microarray genes/probes were filtered prior to analysis by *four* criteria:

1. Mouse genes from scRNA-seq were selected using the noise model from the original work in Zeisel et al. Supplementary Information^8^;
2. The mouse scRNA-seq gene set was reduced using minimum redundancy maximum relevance (mRMR)^40^ to remove highly correlated genes and retain genes with high variance across cell types;
3. Mouse genes from scRNA-Seq were compared to human microarray data to identify homologous matches.
4. Feature selection of concordant human and mouse gene homologs for each pair of mouse gene signature and microarray sample. The gene concordance was evaluated by the rank distribution of the mouse gene list and the microarray samples rank distribution. A specific gene homolog that have similar rank across two gene lists (defined as within a 0.9 standard deviation of z-score distribution) were selected as a feature. The details of each step are discussed in the preprocessing and feature selection section of the supplementary material (Supplementary Information, Sect. 1).

Mouse scRNA-seq data is annotated with more cell types than in human data, so these additional cell types were added to complete the human dataset. These appended mouse cell types “filled in” the missing human cell types.

### Cell-type proportion estimates on simulated scRNA-Seq data

We tested two linear models for cell deconvolution, ordinary least squares regression (OLS) and non-negative matrix factorization (NMFR) on simulated gene expression data from mouse and human brains^6–8^. Only genes that went through the above feature selection protocol were retained in the microarray and scRNA-seq data. For each cell type in the scRNA-seq data, the mean was calculated for each gene in the gene set across all samples of the same cell type. The optimal linear combination of cell types was calculated to reduce the difference with the AHBA microarray vector. NMFR, instead of fitting the optimal linear combination of cell types, fits the optimal linear combination of principal components (taken from cell types), then converts them to cell types by a linear transformation. Specifically, the OLS model predicts proportions of cell types ($\propto$) in a tissue sample ($T$) by fitting a linear combination of cell type expression profiles ($E$) for $c$ cell types.

$$T=\sum_{i=1}^{c}{E}_{i}{\propto }_{i}+\epsilon .$$

In contrast, NMFR estimates the cell type proportions by fitting the same model to the k-component non-negative matrix factorization matrix ($W$) that is calculated from the cell type expression profiles, i.e., $E=WH$. The values in $\beta$ can be converted into corresponding cell type proportions ($\propto$) using the matrix $H$. The detailed description of both regression methods is available in supplemental methods (Supplementary Information, Sect. 2).

$$T=\sum_{i=1}^{k}{W}_{i}{\beta }_{i}+\epsilon .$$

Aside from the two classical statistical methods of deconvolution that we implemented, DWLS was also used in a comparison against our top-performing model. Model prediction consistency with ground truth was determined by comparing the known true proportion of cell types (ground truth) with the cell types predicted by either OLS or NMFR deconvolution model. The deconvolution performance was measured by calculating the PCC between the true proportions and predicted proportions (Fig. 2). Because of the consistent higher agreement with true cell composition, OLS was next compared against a state-of-the-art deconvolution technique called DWLS, which is used for deconvolution of bulk RNA-seq with scRNA-seq. Due the comparable performance of OLS to DWLS and the simplicity of the OLS method (simpler to implement, interpret, requires less computation), we used OLS for further analyses.

### Cell-type proportion estimates across AHBA donors and scRNA-Seq datasets

Cell types identified in mouse data were used to augment missing cell types in human scRNA-seq data. Specifically, if a cell type in the human scRNA-seq was not present, the corresponding cell type from mouse scRNA-seq was added so that each dataset contains all nine major cell types. The resulted scRNA-seq datasets (namely MusNG, HumNG + MusNG, HumN + MusNG) were each used to estimate the proportions of cell types in the AHBA microarray samples.

Then, nine major cell type proportions were deconvoluted for each microarray sample, which was annotated with spatial locations in AHBA. We subsequently compared the consistency of deconvolution results from the three input scRNA-seq datasets. For each pair of comparisons (i.e. MusNG vs. HumN + MusNG, MusNG vs. HumNG + MusNG, HumN + MusNG vs. HumNG + MusNG), the mean PCC from the six brains were computed for each cell type, resulting in 27 total correlations from three pairs of comparisons for nine cell types. P-values and Benjamini–Hochberg false discovery rate (BH-FDR) were also computed for each of the 27 cell-type correlations. With the same procedure, we also compared the difference between the same cell types and mismatched cell types.

The proportion estimates for each cell type were calculated for all three input scRNA-Seq datasets, resulting in three sets of proportions for each of the six AHBA donors, i.e. 18 total cell type proportion estimates. For each of these estimates, the samples within a brain region were then used to create a composite cell-type proportion for that region by taking the average across all samples in that brain region. The PCCs were calculated between each pair of the AHBA donors using these averaged regional proportions for each combination of donors for a given input scRNA-seq dataset. An additional random donor was created by (i) randomly selecting a donor and (ii) randomly reordering that donor’s sample regions. This synthetic data is used to calculate PCC between the random donor and the non-randomized donors. This randomization and correlation process is repeated 100 times and averaged to generate random PCC values as a negative control. The individual correlation matrices for each scRNA-seq dataset were included in the Supplementary Information. The final correlation matrix contains the average PCC value across all three of the scRNA-seq correlation matrices for overall conclusions.

### Cell-type association with specific brain regions

To check how different cell types were localized across brain regions, each of the three scRNA-Seq datasets were used to deconvolute all AHBA brains using OLS. The combination from each input scRNA-Seq dataset and AHBA donor were used for a comprehensive OLS model^41^. All six AHBA brains were manually registered so that the brain regions were consistent across samples. Because some regions and cell types had low representation, the deconvolution outputs (proportions) were smoothed by taking the maximum of the five closest samples in 3D Euclidean space. All figures are the sagittal 2D view of the 3D representation, i.e. using the two dimensions that constitute the saggital view of the brain. The smoothing process improved the coverage of difficult-to-detect cell types.

The color represented the smoothed proportion of each cell type and was mapped to each sample’s respective voxel location in 3D space. The first two dimensions could then be used to generate a 2D visualization. Principle component analysis (PCA) was performed on all cell deconvolution results for all 3702 microarray samples, producing a matrix containing the PCA values for each sample that could be plotted in 2D space. Each point (corresponding to the PCA components of the cell proportion estimate from a single microarray sample) is colored based on the annotated anatomic locations from which that sample was extracted in the donor brain. K-means clustering (k = 3, corresponding to the three major brain regions—cerebrum, brainstem, cerebellum) was applied to cluster the data points into three groups. The consistency between each of the three clusters and the three anatomic regions is measured using sensitivity and specificity values. A MANOVA model was fitted using the sensitivity and specificity as dependent variables and the input scRNA-Seq datasets, AHBA donors, and regions sampled as the independent variables to study the effects of each type of sample on the ability to accurately map back to the tissue of origin. Any significant results for scRNA-Seq dataset, AHBA donor, or brain region correspond to a difference in mapping accuracy due to that variable (i.e., bias).

### Neuron nuclei and mRNA localization discrepancy and adjustment for neural projection volume

The consistency between neuron nuclei location and neuron mRNA localization was evaluated. PCC was computed between the cellular composition results from 18 combinations (six donor brains multiply with 3 different input scRNA-seq data) and the corresponding nuclei count results, and then compare with the PCC from the neuron proportion corrected with neural projection information. To do the latter, we download the mouse brain connectivity data from the Allen Brain Institute to compute the neural projection volume. First, the injection and target site volumes for each experiment as well as the mouse anatomic brain region hierarchy were downloaded. A breath-first-search was employed to extract all regions under the cerebrum, brainstem, and cerebellum branches in the mouse brain region hierarchy. This information was used to stratify the signals to the specified regions. Next, the metadata from each of the AHBA donor brains was used to extract cerebrum, brainstem, and cerebellum samples, stratifying the sample proportions to each region. The ratio of neuron to non-neuron was calculated for each sample and used in subsequent analysis so that the cell-type signature proportions were comparable to the nuclei proportions. Next, all 18 combinations (three input scRNA-Seq datasets on top of six brain donors) were matched to each of the nuclei datasets by brain region such that for each nuclei neuron/non-neuron estimate, there were 18 cell-type signature proportion-derived neuron/non-neuron estimates. Finally, the cell-type expression profile proportion ratios were divided by the axonal projection volumes (mouse connectivity) and a correlation was calculated between nuclei and cell-type expression profile proportions (Fig. 6).

### AD sample estimate and correlation to clinicopathological measurement

RNA-seq samples from MSBB were used to study the established cell type patterns found in AD progression. Using the same process we used for the AHBA samples, our deconvolution method was used to estimate the cell type proportions of Alzheimer’s Disease (AD) and normal brain samples from bulk RNA-seq data in every sample. We performed the same feature selection steps and used the sample-wise z scores to deconvolute the samples in the same manner as the AHBA samples. Next we compared these proportions of cell types to known markers for AD progression. Specifically the tests that we used were the amyloid plaque scale taken during autopsy (Plaque Mean), the Braak stage score (BBS) taken during autopsy, and the clinical dementia rating scale (CDR) via patient interview. Since neurons degenerate while microglia proliferate during AD progression, we calculated the PCC value between these cell types with the three clinicopathological measurements.

## Supplementary information

Supplementary Information.
