# Supplementary material for: Spatial cell type composition in normal and Alzheimers human brains is revealed using integrated mouse and human single cell RNA sequencing
Source: Sci Rep. 2020 Oct 22;10:18014. doi: 10.1038/s41598-020-74917-w (PMC7582925; doi:10.1038/s41598-020-74917-w)
Supplement: Supplementary file 1 — Supplementary Information. [file 41598_2020_74917_MOESM1_ESM.pdf]

Title

Supplementary Materials: Spatial Cell Type Composition in Normal and Alzheimers Human Brains is Revealed Using Integrated Mouse and Human Single Cell RNA Sequencing.

Authors

Travis S Johnson[1,2,3], Shunian Xiang[2], Bryan R. Helm[2], Zachary B Abrams[1], Peter Neidecker[4], Raghu Machiraju[5], Yan Zhang[1], Kun Huang[2,6,7]*, Jie Zhang[7]*

Affiliations

1. Department of Biomedical Informatics, The Ohio State University, Lincoln Tower 250, 1800 Cannon Dr., Columbus, Ohio, 43210
2. Department of Medicine, Indiana University School of Medicine, Emerson Hall 305, 545 Barnhill Dr., Indianapolis, Indiana 46202
3. Department of Biostatistics, Indiana University School of Medicine, HITS 3000, 410 W. 10th St., Indianapolis, Indiana 46202
4. Department of Mathematics, The Ohio State University, Math Tower 100, 231 West 18th Ave., Columbus, Ohio, 43210
5. Department of Computer Science and Engineering, The Ohio State University, Dreese Laboratories 779, 2015 Neil Ave., Columbus, Ohio, 43210
6. Regenstrief Institute, 335, 1101 W. 10th St., Indianapolis, Indiana, 46202
7. Medical and Molecular Genetics, Indiana University Purdue University Indianapolis, HITS 5015, 410 W. 10th St., Indianapolis, Indiana, 46202

*Corresponding authors

Email: jizhan@iu.edu (Zhang J), kunhuang@iu.edu (Huang K).

Running Title: *Johnson et al / Integrative models of spatial cell-type composition*

Funding: This research was supported by a National Institutes of Health NLM-MIDAS Training Fellowship (4T15LM011270-05) to TSJ, National Institutes of Health NLM-NRSA Individual Fellowship (1F31LM013056) to TSJ, and the Indiana University Precision Health Initiative fund to ZJ and KH.

Supplementary Information

Cleaning and Feature Selection

Step 1: Noise Model Filtering by Selection of Genes and Clustering of Mouse Cells

The raw unique molecular identifier (UMI) counts obtained from the MusNG dataset were cleaned and filtered in the same manner as Zeisel et al. [1]. This process took three steps: i) removing low expression genes, ii) removing genes that do not co-express with other genes and iii) selection of top 5000 high variance genes. The intersection of the 5000 MusNG genes with the complete HumNG dataset resulted in 4009 genes that were used in the subsequent analysis. The HumN dataset was not reduced to these 5000 genes because there were too few cell types to perform the mRMR feature selection on this dataset alone.

Step 2: Feature Selection of Genes in scRNA-Seq Data

After filtering, an open-source feature selection algorithm was used to further reduce the number of redundant genes that coexpress within the cortex dataset. mRMR was used to select the top 500 genes from the original 5,000 in the MusNG dataset and 4009 in the HumNG dataset. This tool finds features that are "mutually as dissimilar to each other as possible, but marginally as similar to the classification variable as possible" [2]. This method reduces the number of redundant features that would be included in the cell type models.

Step 3: Matching homologs between Mouse scRNA-Seq and Human AHBA Microarray

After the feature selection step the mRMR-selected genes were then paired with microarray data obtained from AHBA donors individually. The pairing further reduced the number of genes to a subset of genes that were contained in both AHBA and the scRNA-Seq datasets. MusNG contained 423 genes, HumN contained 382 genes, and HumNG contained 62 genes.

Step 4: Selecting Concordant Homologs Between Mouse scRNA-Seq and Human AHBA Microarray

These remaining genes were then center normalized (so that all cell types do not have a higher mean expression across all genes), log2 transformed and converted to a z score. For HumN and HumNG the missing cell types were filled in using the more complete MusNG dataset such that HumN contained 2 human and 7 mouse cell types and HumNG contained 5 human and 3 mouse cell types. Because the AHBA data is fundamentally different due to platform and species genes needed to be selected that behaved the same across the datasets. This introduced a problem in that a traditional correlation matrix method cannot be computed because the microarray and scRNA-Seq samples are not paired by

independent variables. However, gene lists ranked by correct classification of brain regions correlate between mouse and human [3] and mouse and human genes can be selected that will correctly cluster cell types regardless of species[4]. Based on this evidence, we developed a rank based approach using the difference in z-scores of both log2 transformed microarray and scRNA-Seq data matrices to identify concordant homologs. An allowable difference in standard deviation (standard deviation constant) was chosen that corresponded to the maximum allowable change between the mean z score for each gene across all samples.

To attain this, a vector of mean expression (each element for one gene) across all microarray samples was used to compare against the scRNA-Seq data.

$$\bar{T} = \frac{1}{N}\sum_{1}^{N} T_l \qquad \text{(Eq. 1)}$$

Where T_l is expression vector from sample l. Next a vector was generated from the RNA-Seq CTEPs – a weighted average of all CTEPs initialized to a uniform distribution ($\hat{E}$).

$$\hat{E} = Eb^{(j)} \qquad \text{(Eq. 2)}$$

Where $E = \mathbb{R}^{length(G)\times c}$ is the expression RNA-Seq expression matrix (columns are cell type profiles rows are genes) and $b^{(j)}$ contains the weights for each CTEP, $b = \mathbb{R}^{c}\ s.t.\ b_i = \frac{1}{c} \forall\ i \in \mathbb{Z}, 1 \le i \le c$. Variable j represents the iteration of the algorithm such that when first initialized $j = 0$. Then all genes were selected that are within a standard deviation constant of one another.

$$kept \subseteq G\ s.t. \left|\bar{T}^{(g)} - \hat{E}^{(g)}\right| \le d\ \forall\ g \in G \qquad \text{(Eq. 3)}$$

Where $kept$ is the selected gene set, $T \in \mathbb{R}^{length(G)\times N}$ is the microarray expression matrix (columns are samples and rows are genes), G is the set of all genes, d is the standard deviation constant and g is a gene that is within d units of itself between the RNA-Seq and microarray dataset. The predicted proportions ($\hat{b}$) of each CTEP in $\hat{T}$ were calculated using the retained set of concordant gene features ($kept$).

$$\min\left(\left|E^{kept}\hat{b} - \bar{T}^{kept}\right|\right) \qquad \text{(Eq. 4)}$$

$$b^{(j+1)} = z\hat{b} + (1-z)b^{(j)} \qquad \text{(Eq. 5)}$$

Where $z \in \mathbb{R}\ s.t.\ 0 \leq z \leq 1$ is the step size for each iteration of our feature selection algorithm (0.3 was used in this study due to the inflection point in Supplementary Fig. 3). Variable $b^{(j+1)}$ was then incremented with the updated predicted proportions of CTEPS ($\hat{b}$) (eq. 5) then j was incremented ($j = j + 1$). The error was then calculated.

$$error = \left| E^{kept}\hat{b} - \bar{T}^{kept} \right| \quad \text{(Eq. 6)}$$

These steps (2-5) were repeated until there was no longer a decrease in the error between steps.

The standard deviation constant (d) and step size (z) have unique features that can have effects on the estimates (Supplementary Fig. 3). High standard deviation constants correspond to more genes that have a lower correlation between the datasets and low standard deviation constants correspond to fewer genes that have a higher correlation between the microarray and scRNA-Seq datasets. The standard deviation constant was set to 0.9 for most of the analysis and step size to 0.3 due to the inflection point shown in (Supplementary Fig. 3).

Deconvolution Techniques

Ordinary Least Squares Regression (OLS)

The tissue level expression can be thought of as the weighted mean of the cell types within that sample [5-7]. Eq 7, the linear combination of c cell types within the tissue sample T_l, easily represents this.

$$T_l = \sum_{i=1}^{c} E_i \propto_i + \epsilon \quad \text{(Eq 7.)}$$

Where α_i is the proportion ($0 \leq \propto_i \leq 1, \sum_{i=1}^{c} \propto_i = 1, i \in \mathbb{Z}, 1 \leq i \leq c$) of cell type i in the tissue sample T_l, E_i is the expression profile of cell type i and ϵ is the error vector. This can be re-written as Eq 8.

$$T_l = E \propto +\varepsilon, E \in \mathbb{R}^{kept \times c}, \propto \in \mathbb{R}^c \quad \text{(Eq 8.)}$$

A MATLAB script was used to fit a linear model to each of the samples within each AHBA donor using each of the three scRNA-Seq dataset derived CTEPs. The MATLAB function lsqnonneg was used to calculate the α values from the mean expression levels across the retained genes that were selected for the cell type model in each of the tissue samples. The ordinary least squares regression (OLS) method does not include a feature reduction step, which in some applications could be detrimental (e.g., clustering). However, due to the filtering

of redundant features in the dataset using mRMR much of the feature reduction had already been performed.

Non-negative Matrix Factorization Regression (NMFR)

NMFR is based on Principal Components Regression (PCR), an alternative to OLS, which takes advantage of existing variance in the data by fitting new components to the data that maximize the data variance [8]. In the case of PCR, a score matrix (S) and loading matrix (P) is calculated from E (the matrix of concatenated $E_i, i \in \mathbb{Z}, 1 \leq i \leq c$) such that $S_j$is the jth principle component of the model—the component with the jth highest variance. Regression can be run on the principle components (score matrix S).

$$T_l = S\beta + \varepsilon, S \in \mathbb{R}^{kept \times r}, \beta \in \mathbb{R}^r \quad \text{(Eq 9.)}$$

Where β is the weight of principle component j in the linear combination of principle components in T_l, while S_j is the expression profile of principle component j. To retrieve the cell type specific proportions ($\propto$) from the PCR, β can be transformed back into the original variables ($\propto$) using the loading matrix P.

$$\propto = \beta^T P, P \in \mathbb{R}^{r \times kept} \quad \text{(Eq 10.)}$$

However, the main problem encountered when using PCR to deconvolute cell types is that the proportion of each cell type is not by definition non-negative. To avoid this problem, non-negative matrix factorization (NMF) can be used instead to create a lower dimension matrix from the original cell type matrix with c columns, corresponding to each CTEP and kept rows corresponding to the selected features (genes). NMF is a technique where a matrix can be approximated into the product of two non-negative factor matrices [9,10]. Let E be the raw expression matrix.

$$E \approx WH, E \in \mathbb{R}^{kept \times c}, W \in \mathbb{R}^{kept \times r}, H \in \mathbb{R}^{r \times c} \quad \text{(Eq 11.)}$$

The non-negative matrix factorization of E produces W and H which can be thought of as the non-negative score matrix and loading matrix respectively. By treating the non-negative approximation matrix W as the principal components and the non-negative fit function used in the OLS model, non-negative β values are produced that can then be used as estimates of cell type densities. Eq 9 and 10 can be rewritten in terms of W and H to attain non-negative estimates.

$$T_l = W\beta + \varepsilon \quad \text{(Eq 12.)}$$

$$\propto = \beta^T H \quad \text{(Eq 13.)}$$

Significance testing of cell type correlations

To test whether the predicted cell type proportions were consistent in their spatial distribution regardless of the starting dataset (MusNG, HumN, HumNG), we calculated correlations and performed significance testing. As described in the main text we calculated the correlation of each cell type to itself across starting datasets. These correlations were used in the main text Fig. 3. We also calculated every combination of cell types within dataset and across datasets for each starting dataset comparison. This resulted in a correlation matrix where all cell type correlations could be studied (Supplementary Fig. 4-10). Since the overall cross dataset correlations are of interest we also display the mean correlation across all brains for each of the three comparisons (MusNG+HumN, MusNG+HumNG, HumN+HumNG, Supplementary Fig. 4). We find that in this overall comparison the same cell type comparisons are always more positively correlated than mismatched cell type correlations (Supplementary Table I).

Principal cell types visualized across the entire AHBA

To show high-level spatial distributions of principal cell types, all six brains across all cell types are combined into single 3D representations for each of the three scRNA-Seq datasets. SVD was performed on the cell-type proportion matrix, and the three largest components were used as the principal cell types across all samples in each brain to display the results as a three-digit color vector. This analysis is performed for each of the six AHBA brains using each of the three scRNA-Seq datasets individually. The principal cell types are displayed in 3D using color to represent the top three principal cell types. The three scRNA-Seq datasets are displayed separately such that all six AHBA brains are manually overlaid for each scRNA-Seq dataset. This registration produces consistent anatomic locations across the six overlaid brains. Displays are generated with MATLAB function scatter3 (The Mathworks, Inc.; Natick, MA, USA) to view sagittal, coronal, and axial images.

To visualize the structural patterns among estimated cell types, we applied singular value decomposition (SVD) to estimated cell-type data from the AHBA to reduce each mapped cell type to three principal types, which then could be displayed in an R^3 color vector. The 3D output for each of the six brains was overlaid by anatomic location. This visualization showed that there were unique patterns associated with cerebrum, brainstem, and cerebellum brain regions (Figure 7A-I). For example, the cerebrum displays a cell-type pattern that is

distinct from that of both the brainstem and cerebellum. In contrast, although the brainstem and cerebellum exhibited differences from one another, the cell types within these two regions were similar (Figure 7A-I). These patterns were consistent across brains and among the input scRNA-Seq datasets that were used to deconvolute the samples (Figure 7A-I).

We also evaluated the principal cell types in each major brain region visually (Figure 7J-L, Supplementary Table III). The brainstem was generally comprised of CA1 pyramidal and glial cell types. The cerebellum was comprised of interneurons and various glial cell types (Figure 7J-L, Supplementary Table III). Though detailed information on specific cell-type locations are not common, it is worth noting that some patterns are consistent with known locations; for example, ependymal cells localized to the spinal cord and ventricular regions.

Supplementary Figures

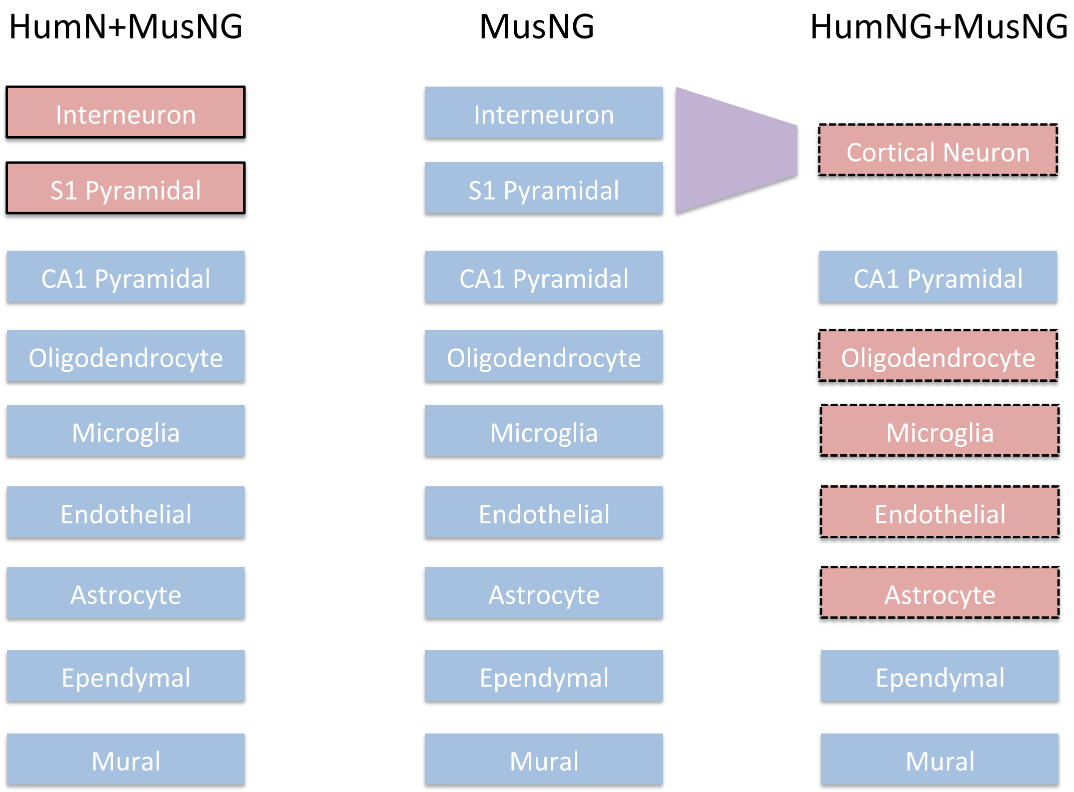

Supplementary Figure 1 To have comparable datasets some cell types had to be used from the mouse dataset to complete the two human datasets. Red boxes indicate human cell types and blue indicate mouse cell types. The original HumN cell types are outlined in a solid line. The original MusNG cell types have no outline. The original HumNG cell types are outlined in a dashed line.

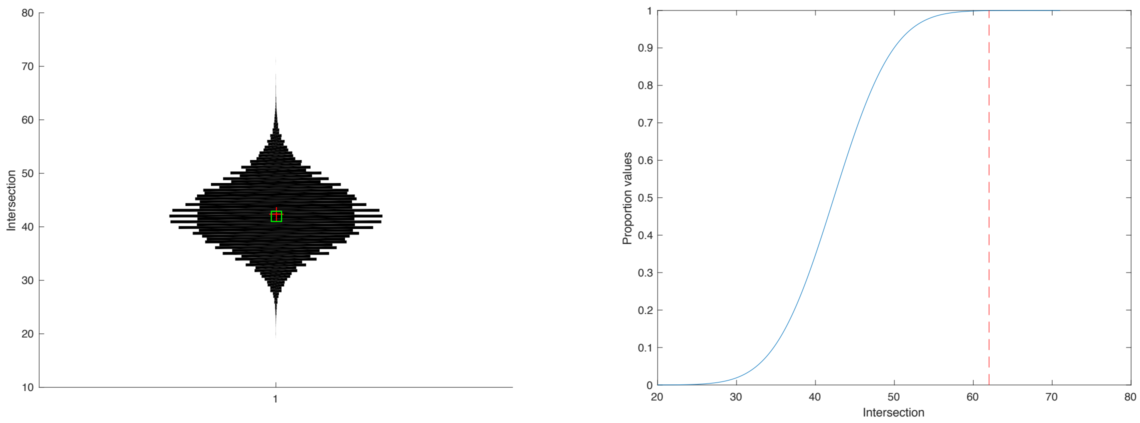

Supplementary Figure 2 This plot represents the empirical study of the likelihood of acquiring an intersection of 62 features from 2 randomly selected samples described in the discussion section. Left is the distribution of intersections between two randomly selected datasets. Right is the CDF of that distribution with the intersection found during our study marked by the red dashed line.

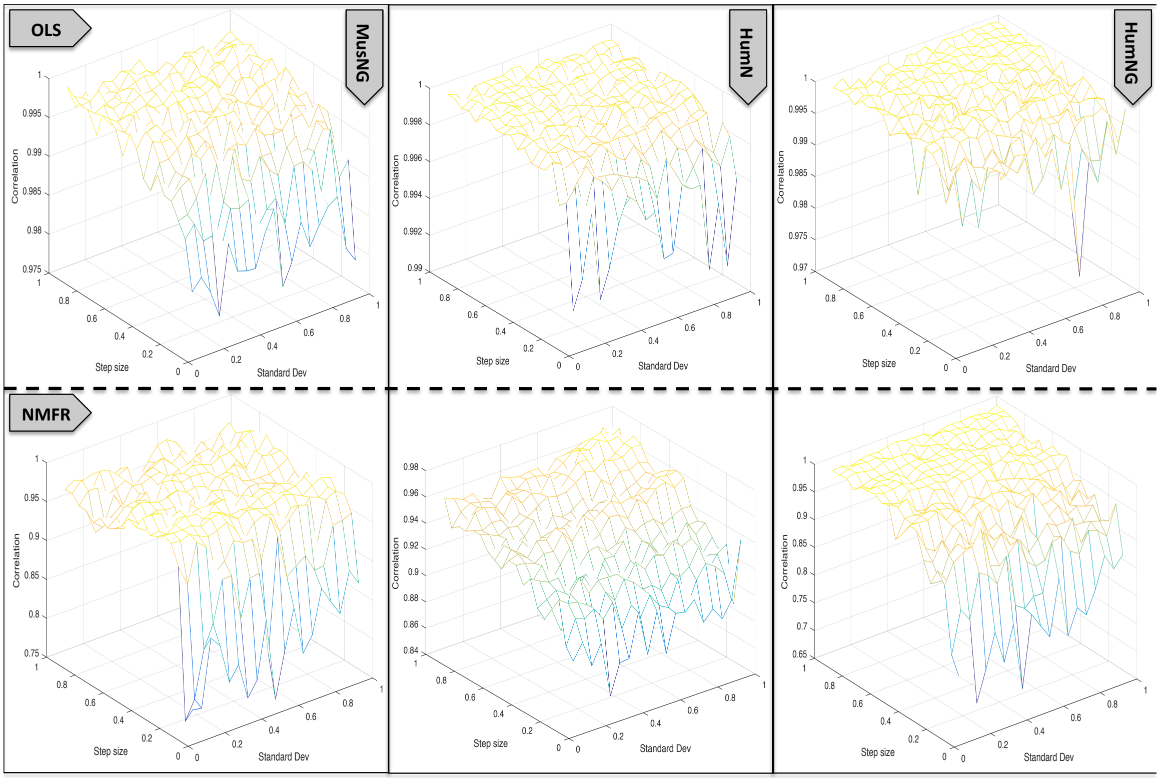

Supplementary Figure 3 Each plot represents the changes in correlation between the estimated and true cell type proportions with the change in step size (z Eq. 5) and (d Eq. 3). The rows represent the algorithm used to estimate the proportions and the columns represent the scRNA-Seq datasets used to generate the artificial sample and cell type expression profiles.

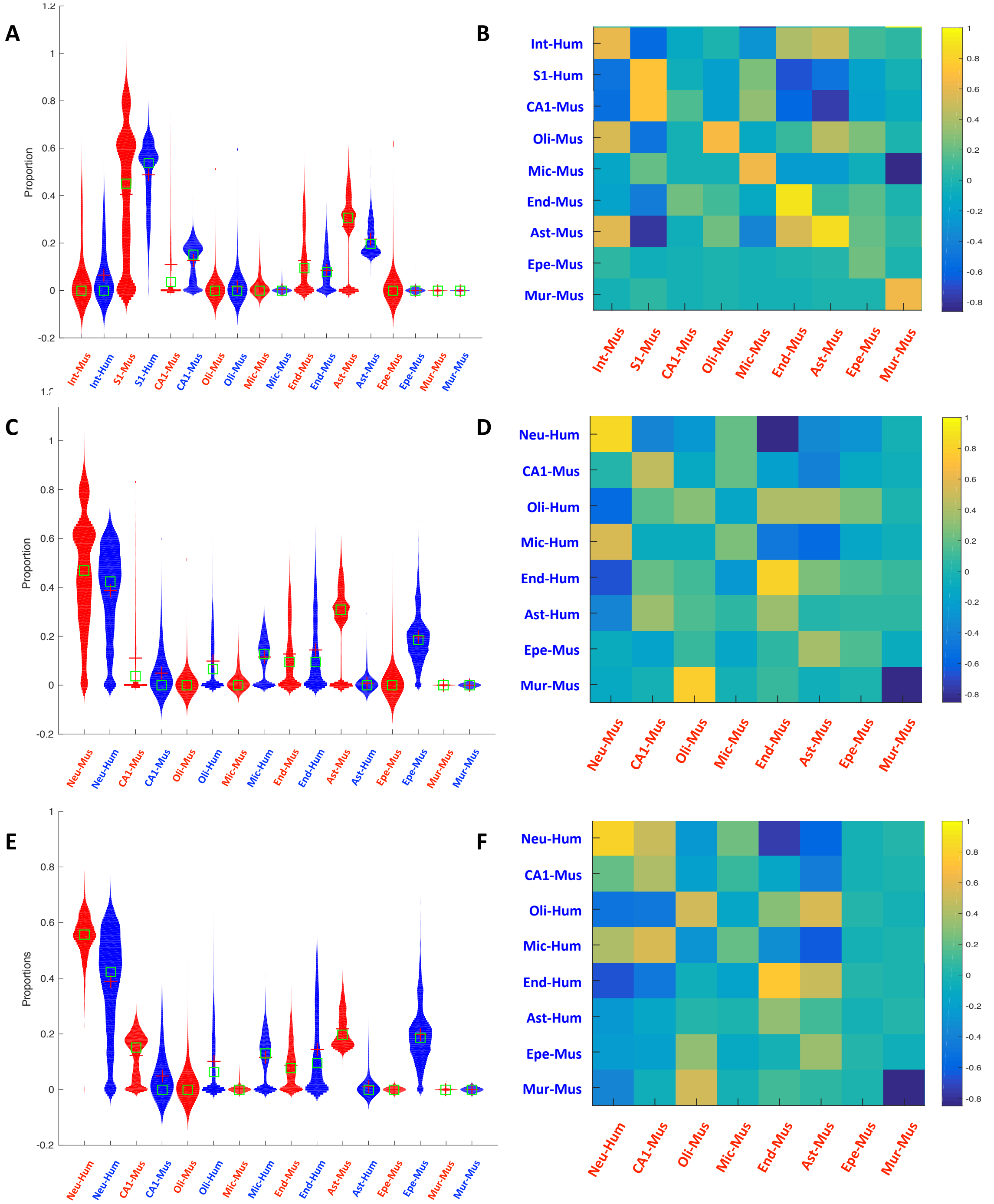

Supplementary Figure 4 Consistency of cell type proportions using each of the scRNA-Seq datasets to deconvolute the AHBA. Each figure is the combinations of all 6 AHBA donors. **A)** Violin plot of MusNG (red) and HumN (blue) distributions of cell type proportions for each cell type. **B)** The cell proportion correlations for each cell type originated from MusNG (blue) vs. from HumN (red) **C)** Violin plot of MusNG (red) and HumNG (blue) distributions of cell type proportions for each cell type. **D)** The cell proportion correlations for each cell type originated from MusNG and HumNG . **E)** Violin plot of HumN (red) and HumNG (blue) distributions of cell type proportions for each cell type. **F)** The cell proportion correlations for each cell type originated from HumN and HumNG . **B,C,D)** The red fonts correspond to the red distribution and the blue fonts to the blue distribution plots in **A,C,E**.

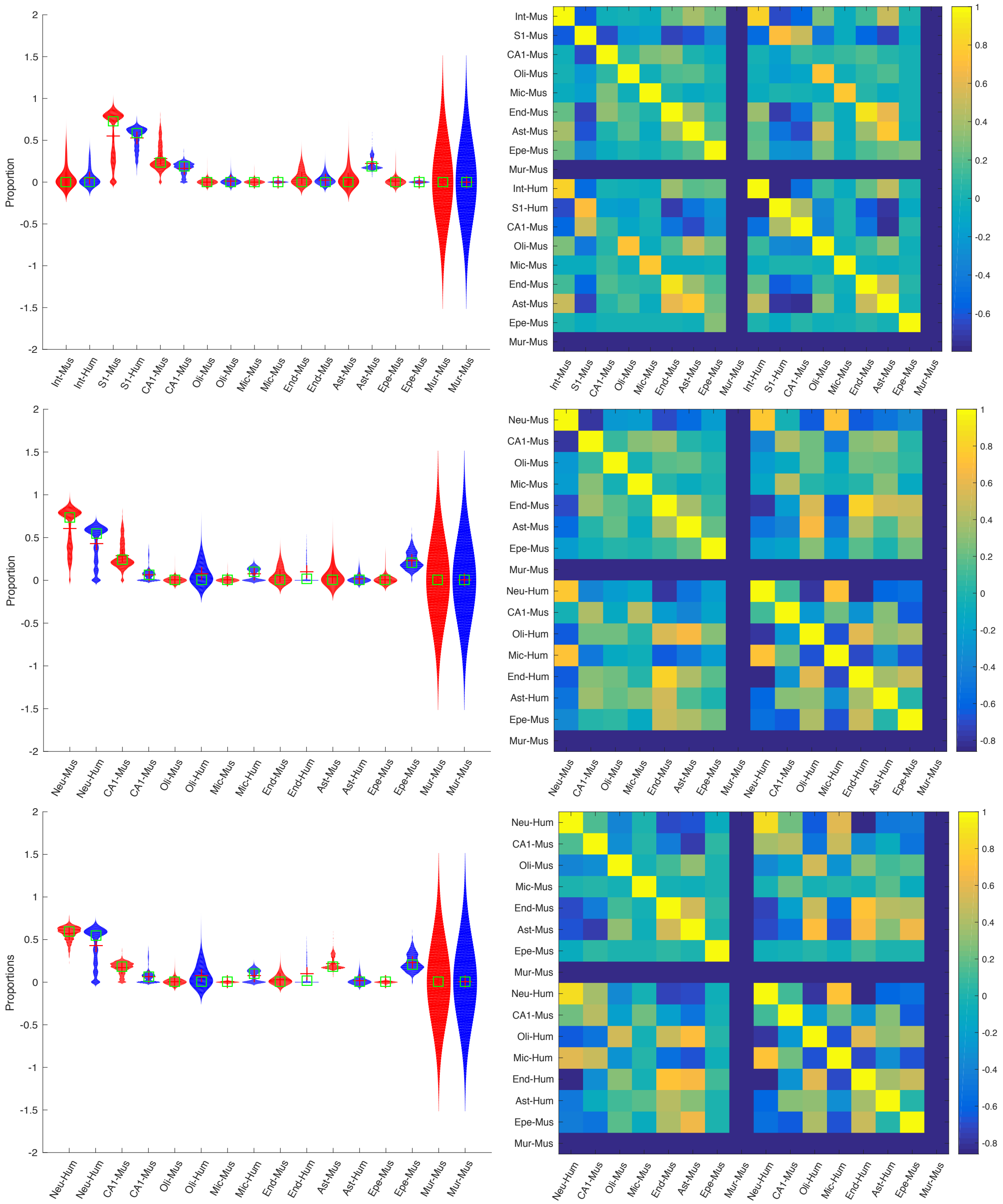

Supplementary Figure 5 Comparison of cell type distributions between each combination of datasets within donor 9861. Top: MusNG (red) and HumN (blue), Middle: MusNG (red) and HumNG (blue), Bottom: HumN (red) and HumNG (blue). Left column are the cell type distributions from donor 9861 such that each cell type signature distribution (x axis) contains every observed proportion for that cell type across all samples in donor 9861's brain. Right column are the spatial correlations between cell type proportions between datasets (p-values in Supplementary Table 2).

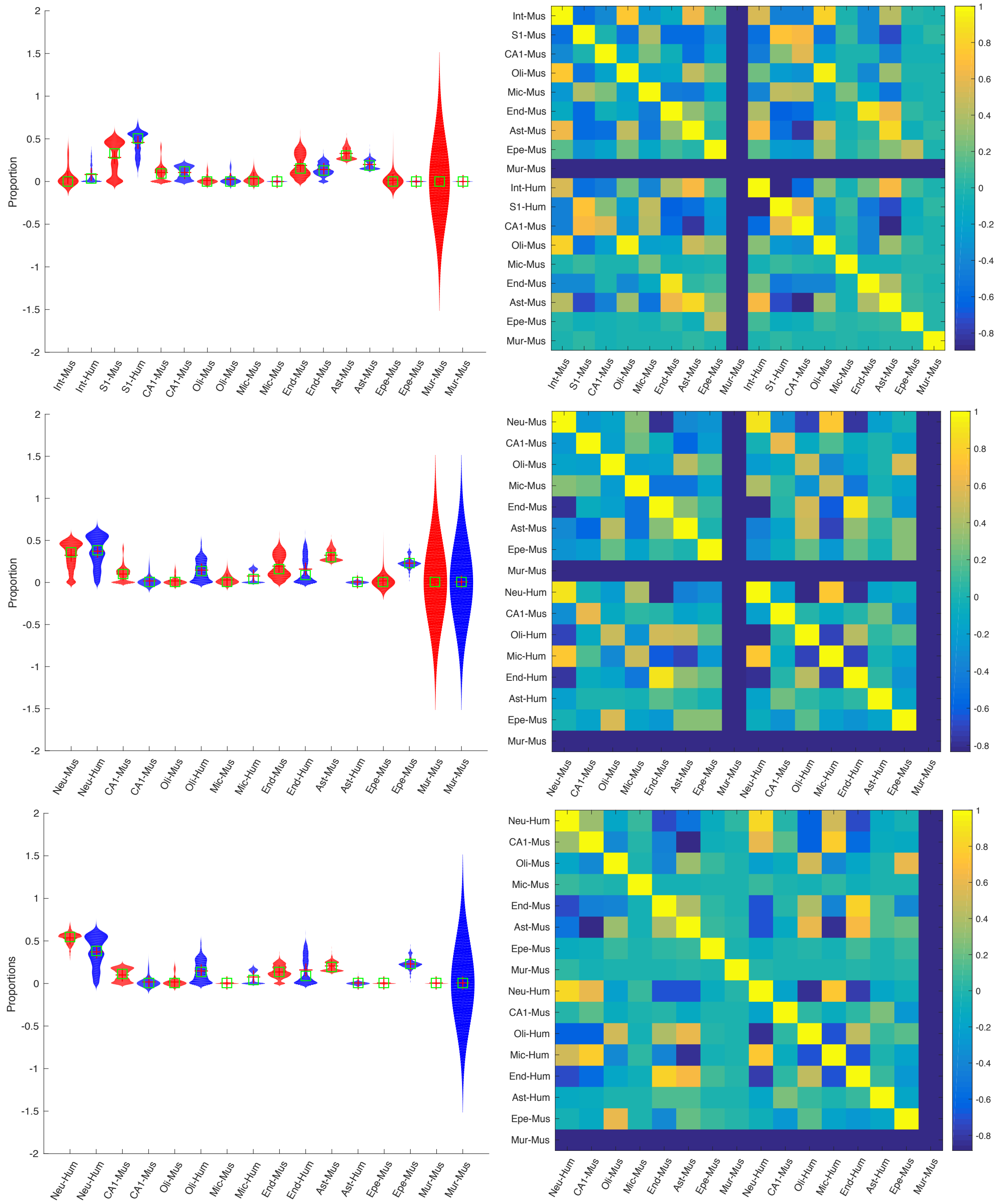

Supplementary Figure 6 Comparison of cell type distributions between each combination of datasets within donor 10021. Top: MusNG (red) and HumN (blue), Middle: MusNG (red) and HumNG (blue), Bottom: HumN (red) and HumNG (blue). Left column are the cell type distributions from donor 10021 such that each cell type signature distribution (x axis) contains every observed proportion for that cell type across all samples in donor 10021's brain. Right column are the spatial correlations between cell type proportions between datasets (p-values in Supplementary Table 2).

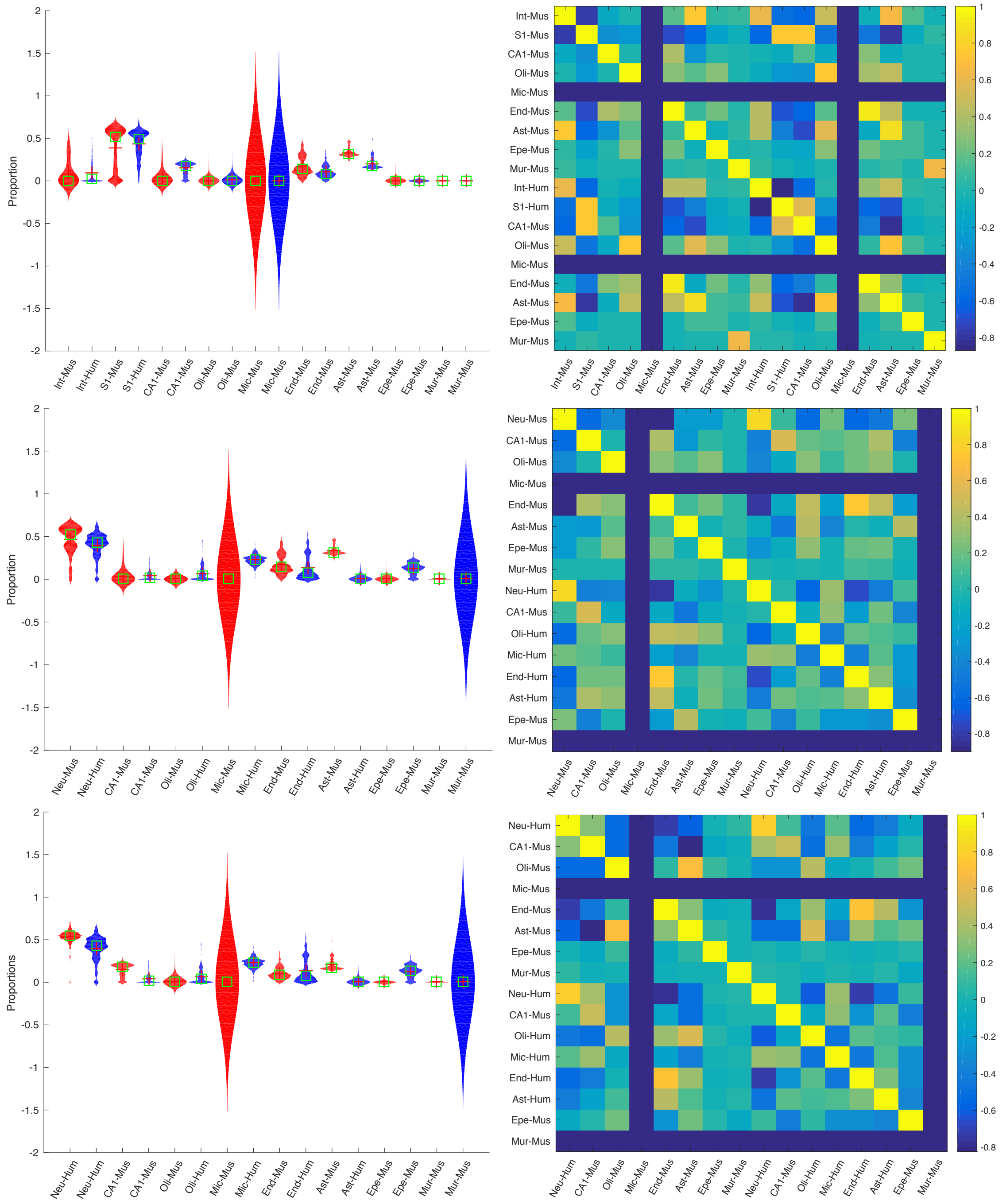

Supplementary Figure 7 Comparison of cell type distributions between each combination of datasets within donor 12876. Top: MusNG (red) and HumN (blue), Middle: MusNG (red) and HumNG (blue), Bottom: HumN (red) and HumNG (blue). Left column are the cell type distributions from donor 12876 such that each cell type signature distribution (x axis) contains every observed proportion for that cell type across all samples in donor 12876's brain. Right column are the spatial correlations between cell type proportions between datasets(p-values in Supplementary Table 2).

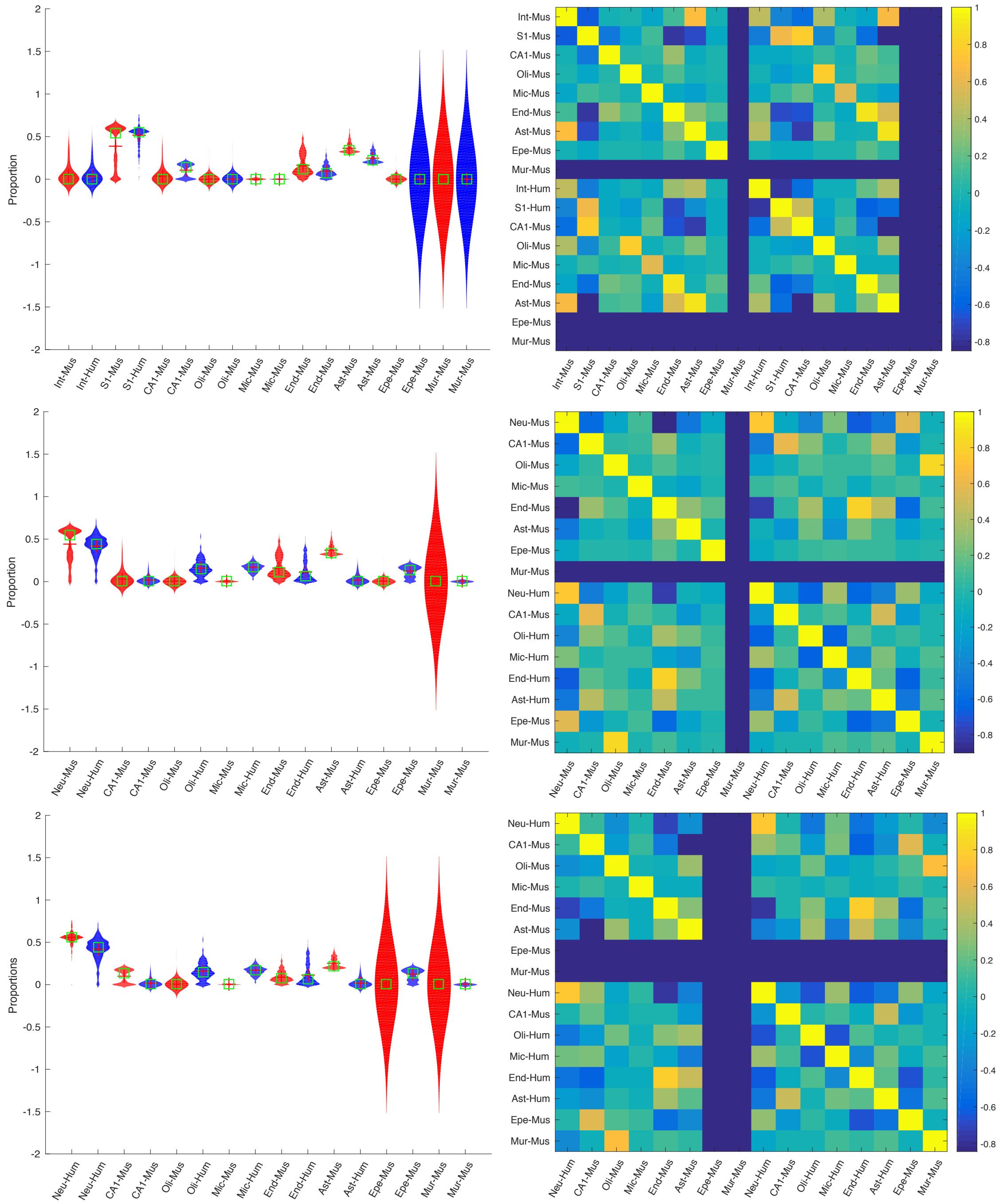

Supplementary Figure 8 Comparison of cell type distributions between each combination of datasets within donor 14380. Top: MusNG (red) and HumN (blue), Middle: MusNG (red) and HumNG (blue), Bottom: HumN (red) and HumNG (blue). Left column are the cell type distributions from donor 14380 such that each cell type signature distribution (x axis) contains every observed proportion for that cell type across all samples in donor 14380's brain. Right column are the spatial correlations between cell type proportions between datasets (p-values in Supplementary Table 2).

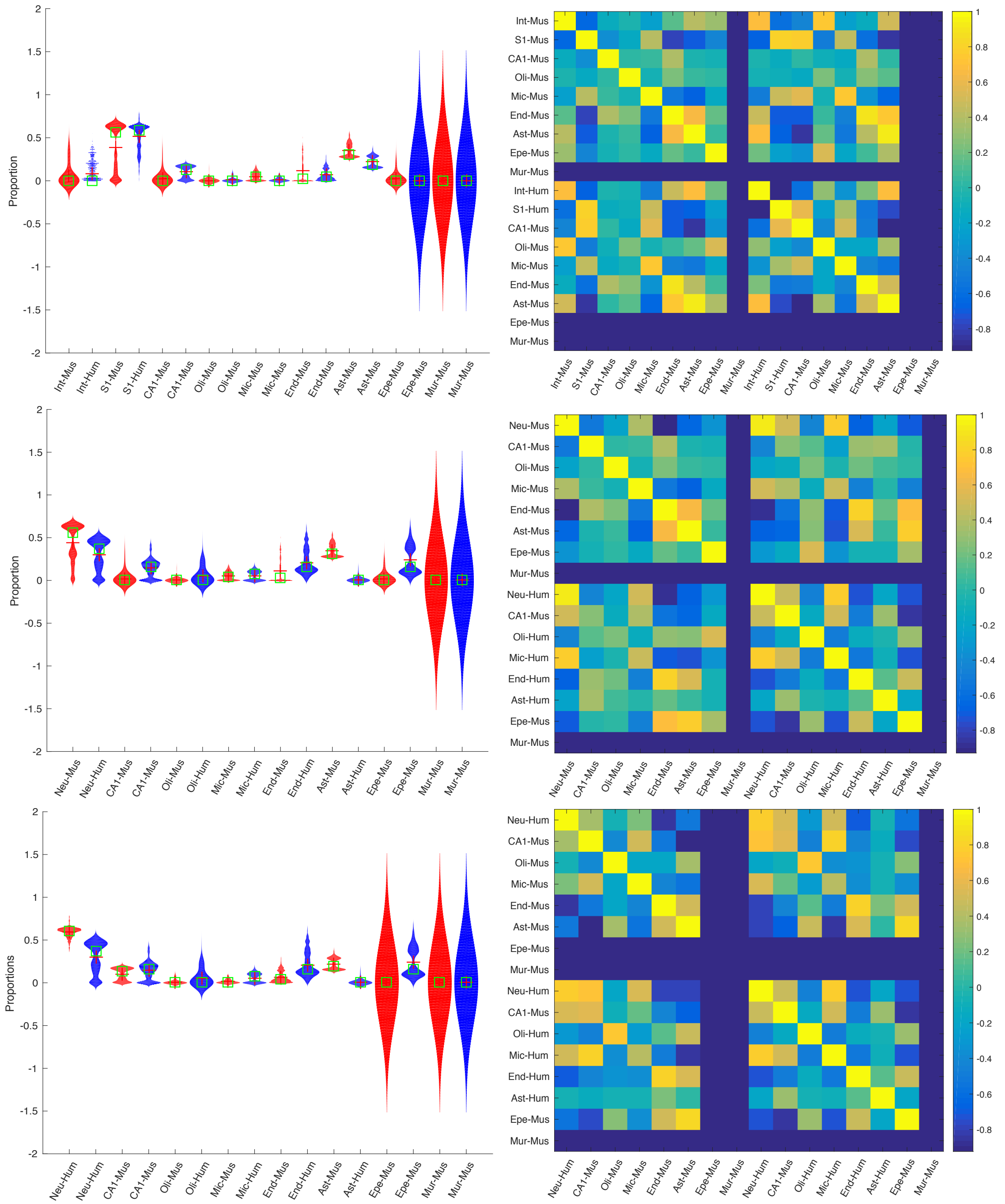

Supplementary Figure 9 Comparison of cell type distributions between each combination of datasets within donor 15496. Top: MusNG (red) and HumN (blue), Middle: MusNG (red) and HumNG (blue), Bottom: HumN (red) and HumNG (blue). Left column are the cell type distributions from donor 15496 such that each cell type signature distribution (x axis) contains every observed proportion for that cell type across all samples in donor 15496's brain. Right column are the spatial correlations between cell type proportions between datasets (p-values in Supplementary Table 2).

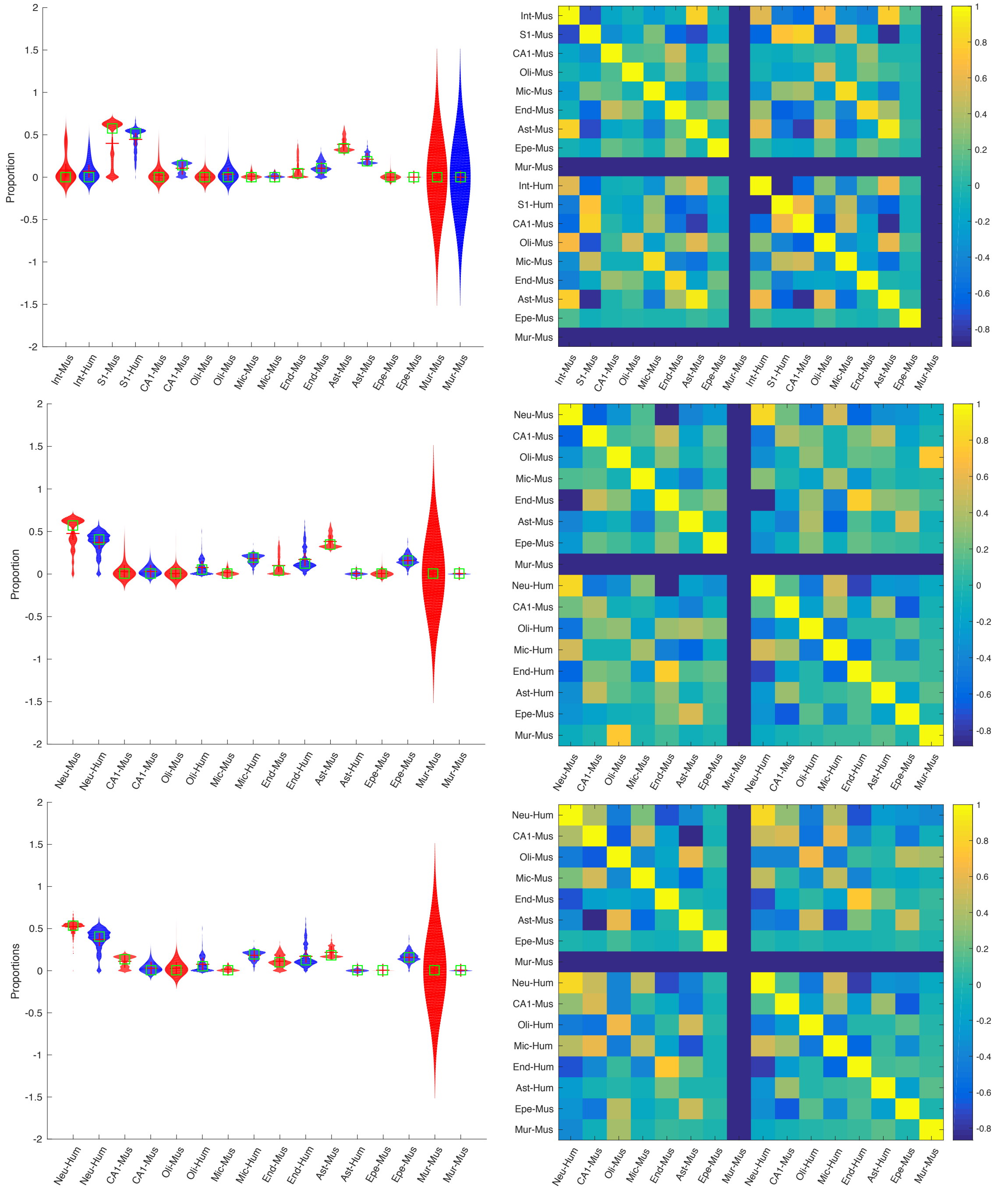

Supplementary Figure 10 Comparison of cell type distributions between each combination of datasets within donor 15697. Top: MusNG (red) and HumN (blue), Middle: MusNG (red) and HumNG (blue), Bottom: HumN (red) and HumNG (blue). Left column are the cell type distributions from donor 15697 such that each cell type signature distribution (x axis) contains every observed proportion for that cell type across all samples in donor 15697's brain. Right column are the spatial correlations between cell type proportions between datasets (p-values in Supplementary Table 2).

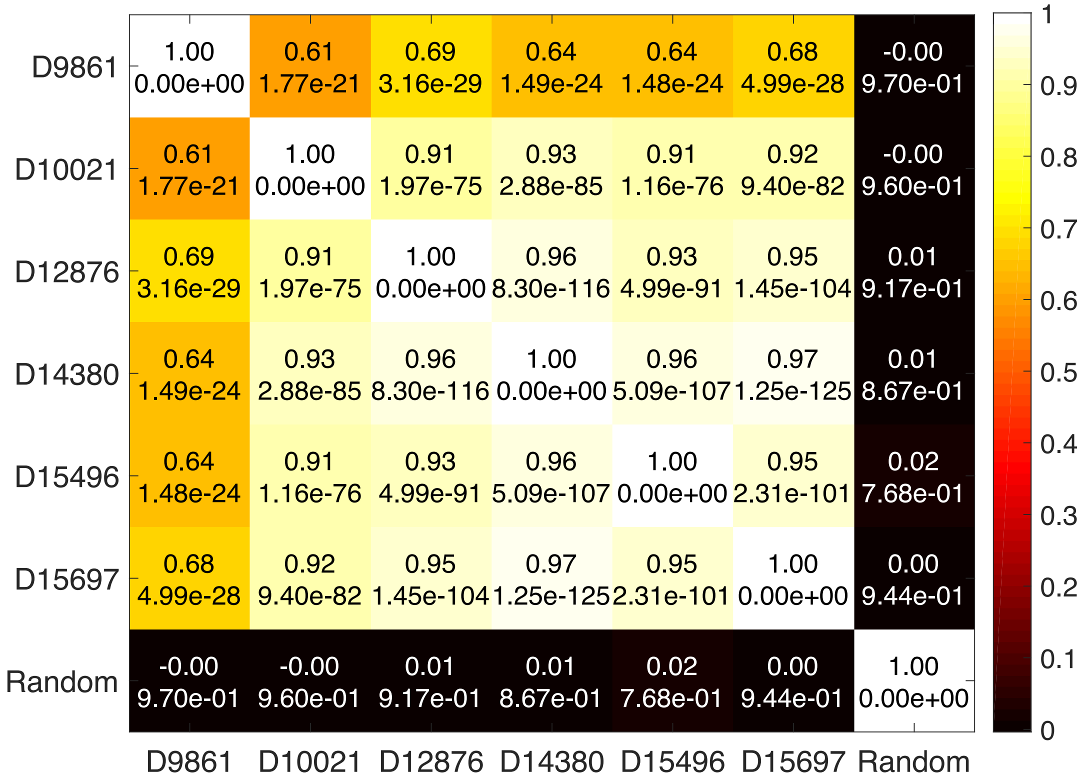

Supplementary Figure 11 Donor to donor comparison using MusNG expression signatures. The cell type distributions across all 6 brains were compared by pooling the samples into overlapping anatomic locations. The random dataset constitutes a five-fold randomization of the sample labels such that it reflects the background noise and spurious correlation. Each row and column are a brain donor. Anova p-value between random and non-random donors is 1.1799×10^{-23}.

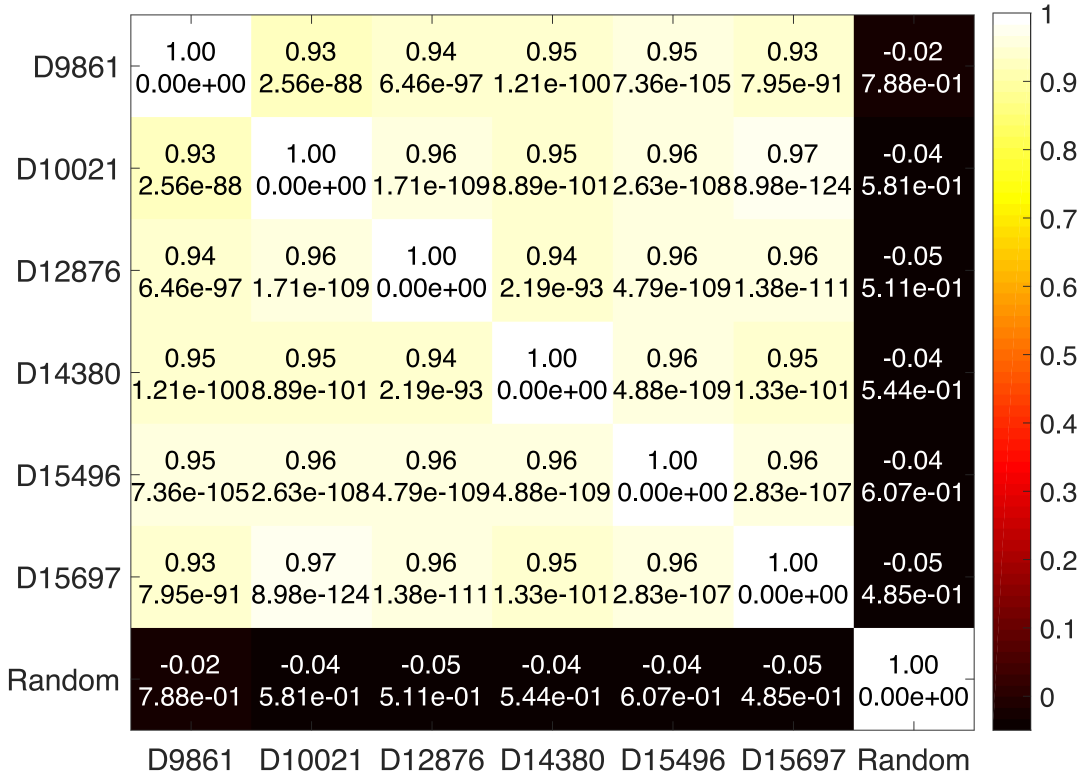

Supplementary Figure 12 Donor to donor comparison using HumN expression signatures. The cell type distributions across all 6 brains were compared by pooling the samples into overlapping anatomic locations. The random dataset constitutes a five-fold randomization of the sample labels such that it reflects the background noise and spurious correlation. Each row and column are a brain donor. Anova p-value between random and non-random donors is 1.5364×10^{-27}.

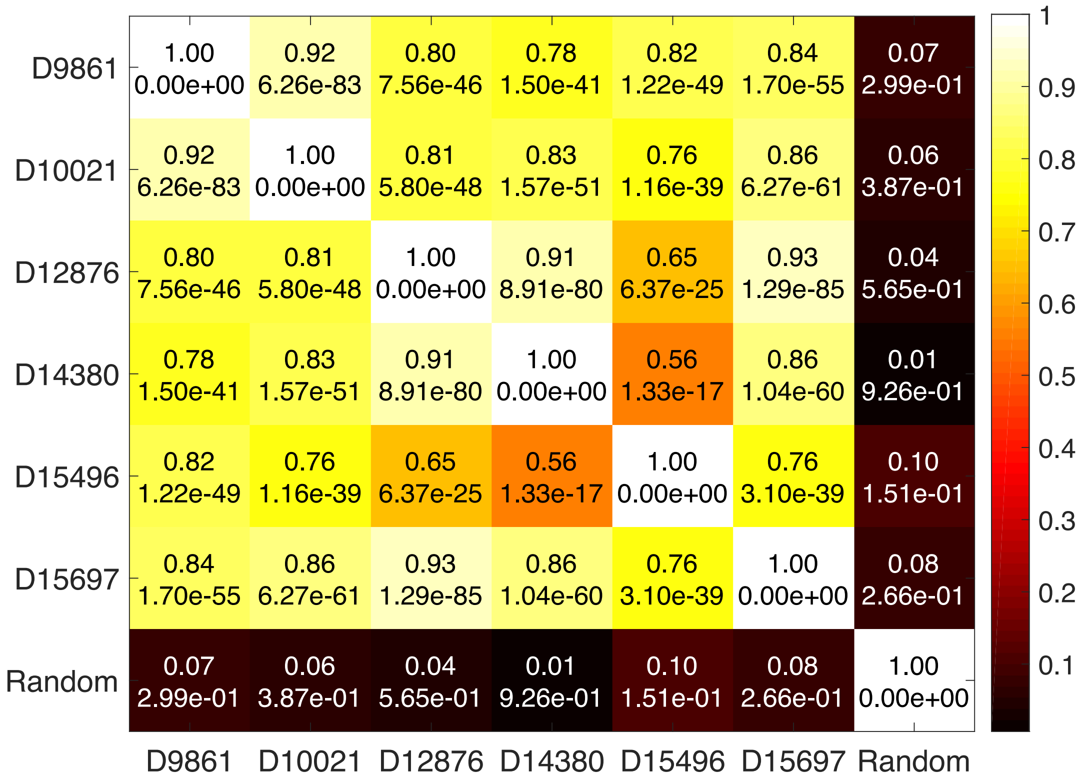

Supplementary Figure 13 Donor to donor comparison using HumNG expression signatures. The cell type distributions across all 6 brains were compared by pooling the samples into overlapping anatomic locations. The random dataset constitutes a five-fold randomization of the sample labels such that it reflects the background noise and spurious correlation. Each row and column are a brain donor. Anova p-value between random and non-random donors is 2.1421×10^{-11}.

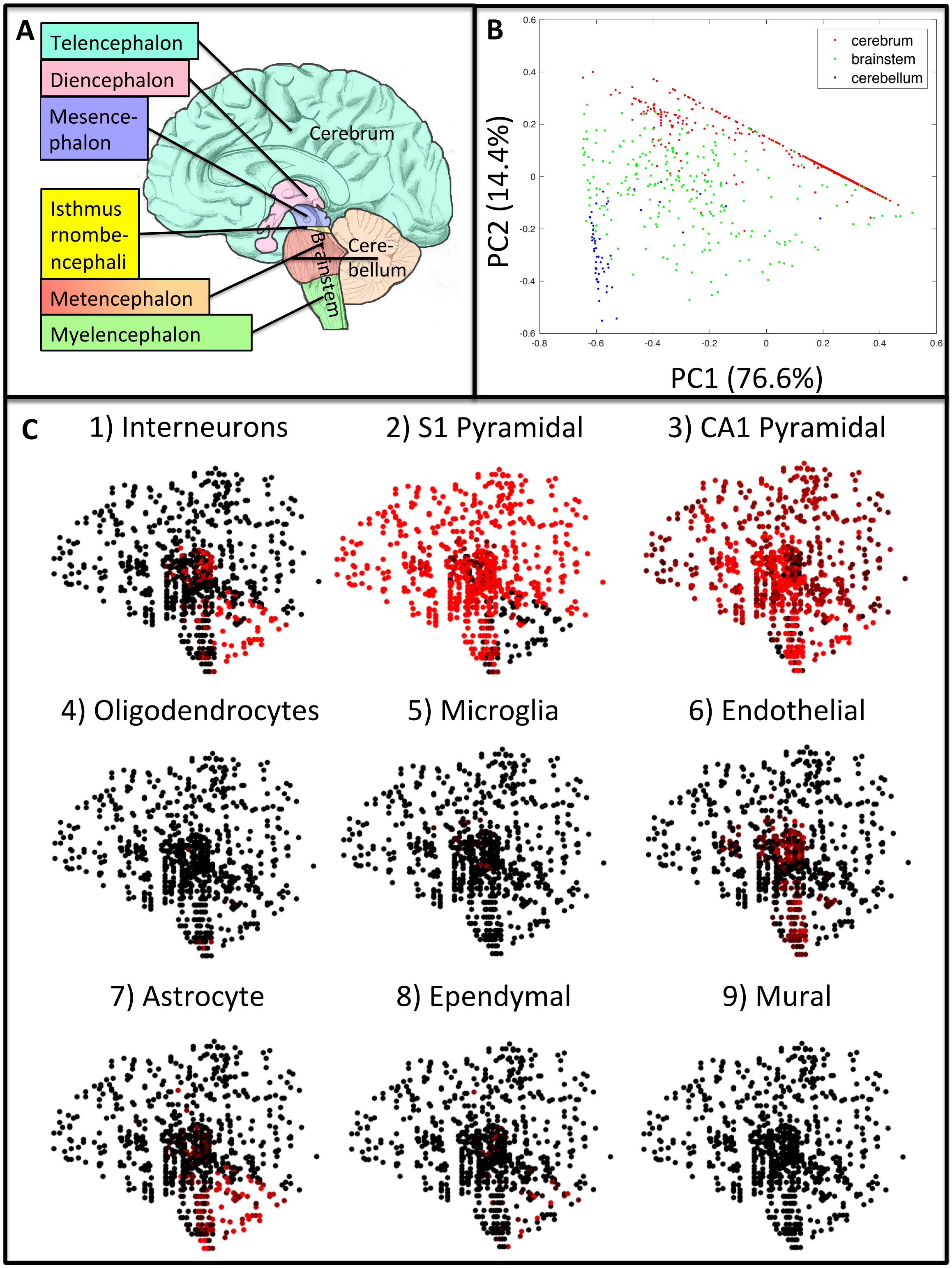

Supplementary Figure 14 Example of spatial distributions of cell type proportions in AHBA donor 9861 using MusNG to deconvolute the RNA expression profile. **A)** A reference map with developmental structures and major brain regions marked. **B)** PCA plot of the cell type proportion matrix such that the 9 cell types are reduced to 2 principal cell types for each of the samples in the brain. The colors indicate the

three regions (cerebrum, brainstem, and cerebellum). **C)** The proportion of each cell type plotted from a sagittal view; Red: high cell proportion (1); Black: low cell proportion (0) on a scale 0-1.

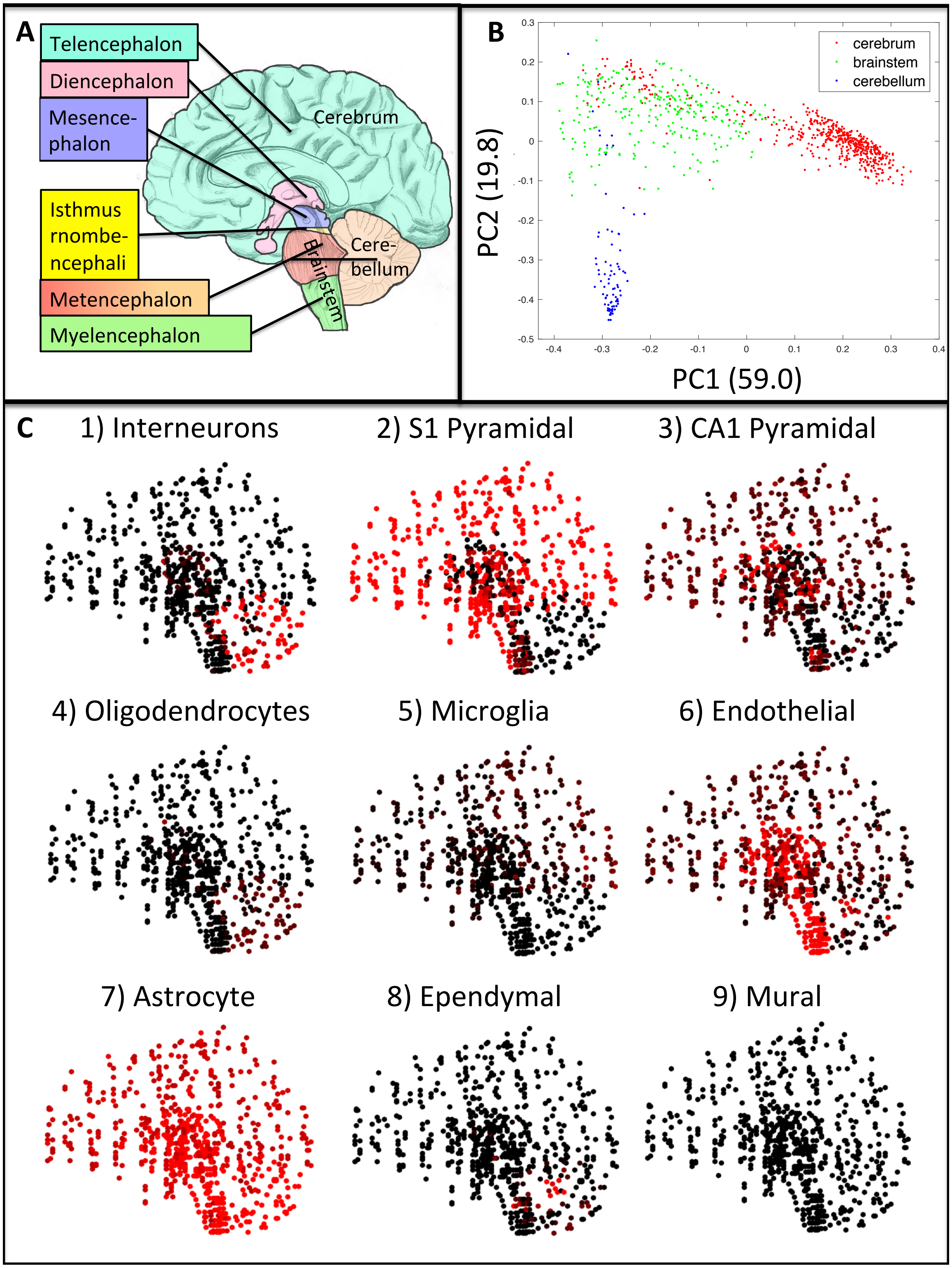

Supplementary Figure 15 Example of spatial distributions of cell type proportions in AHBA donor 10021 using MusNG to deconvolute the RNA expression profile. **A)** A reference map with developmental structures and major brain regions marked. **B)** PCA plot of the cell type proportion matrix such that the 9

cell types are reduced to 2 principal cell types for each of the samples in the brain. The colors indicate the three regions (cerebrum, brainstem, and cerebellum). **C)** The proportion of each cell type plotted from a sagittal view; Red: high cell proportion (1); Black: low cell proportion (0) on a scale 0-1.

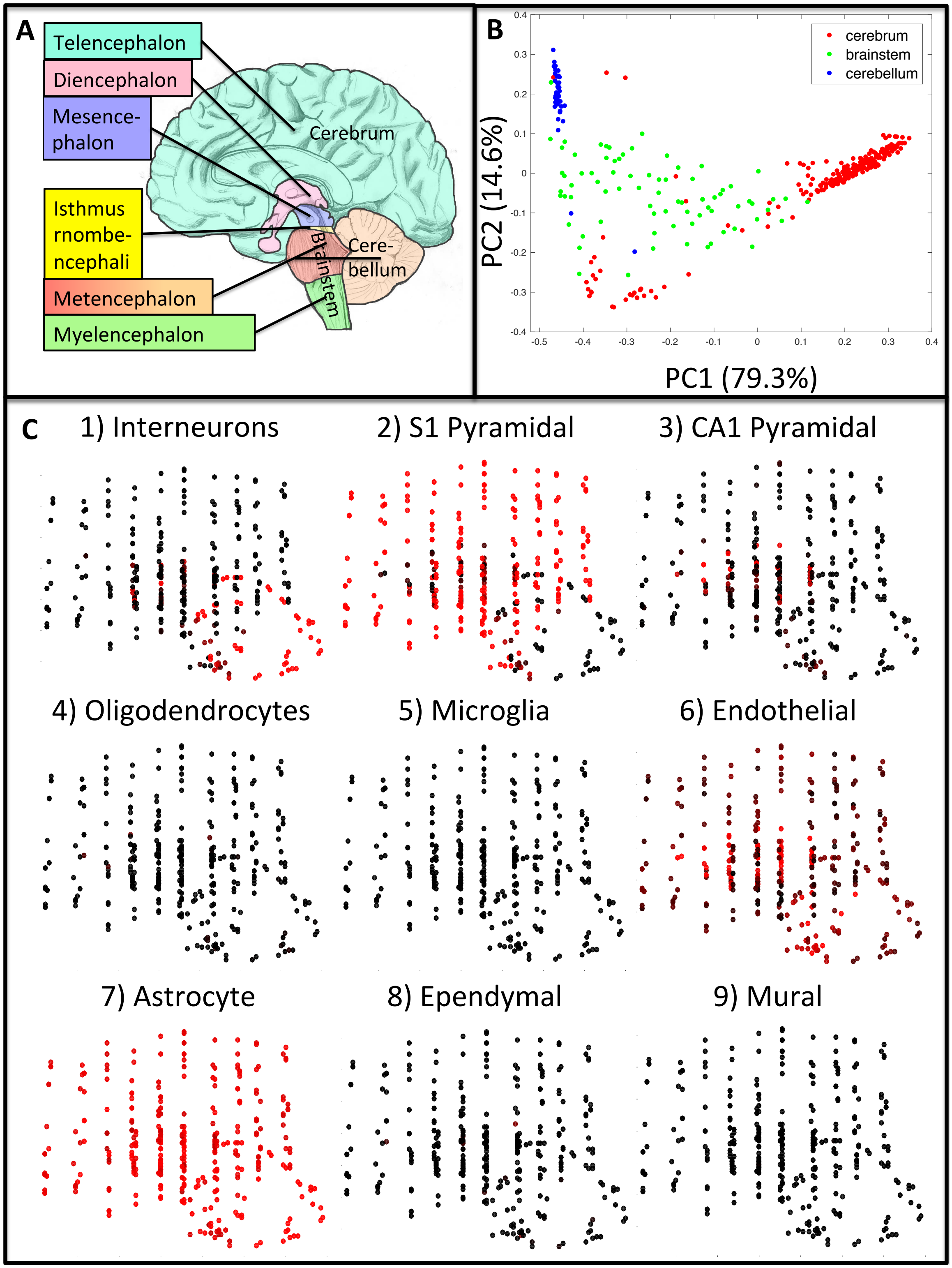

Supplementary Figure 16 Example of spatial distributions of cell type proportions in AHBA donor 12876 using MusNG to deconvolute the RNA expression profile. **A)** A reference map with developmental

structures and major brain regions marked. **B)** PCA plot of the cell type proportion matrix such that the 9 cell types are reduced to 2 principal cell types for each of the samples in the brain. The colors indicate the three regions (cerebrum, brainstem, and cerebellum). **C)** The proportion of each cell type plotted from a sagittal view; Red: high cell proportion (1); Black: low cell proportion (0) on a scale 0-1.

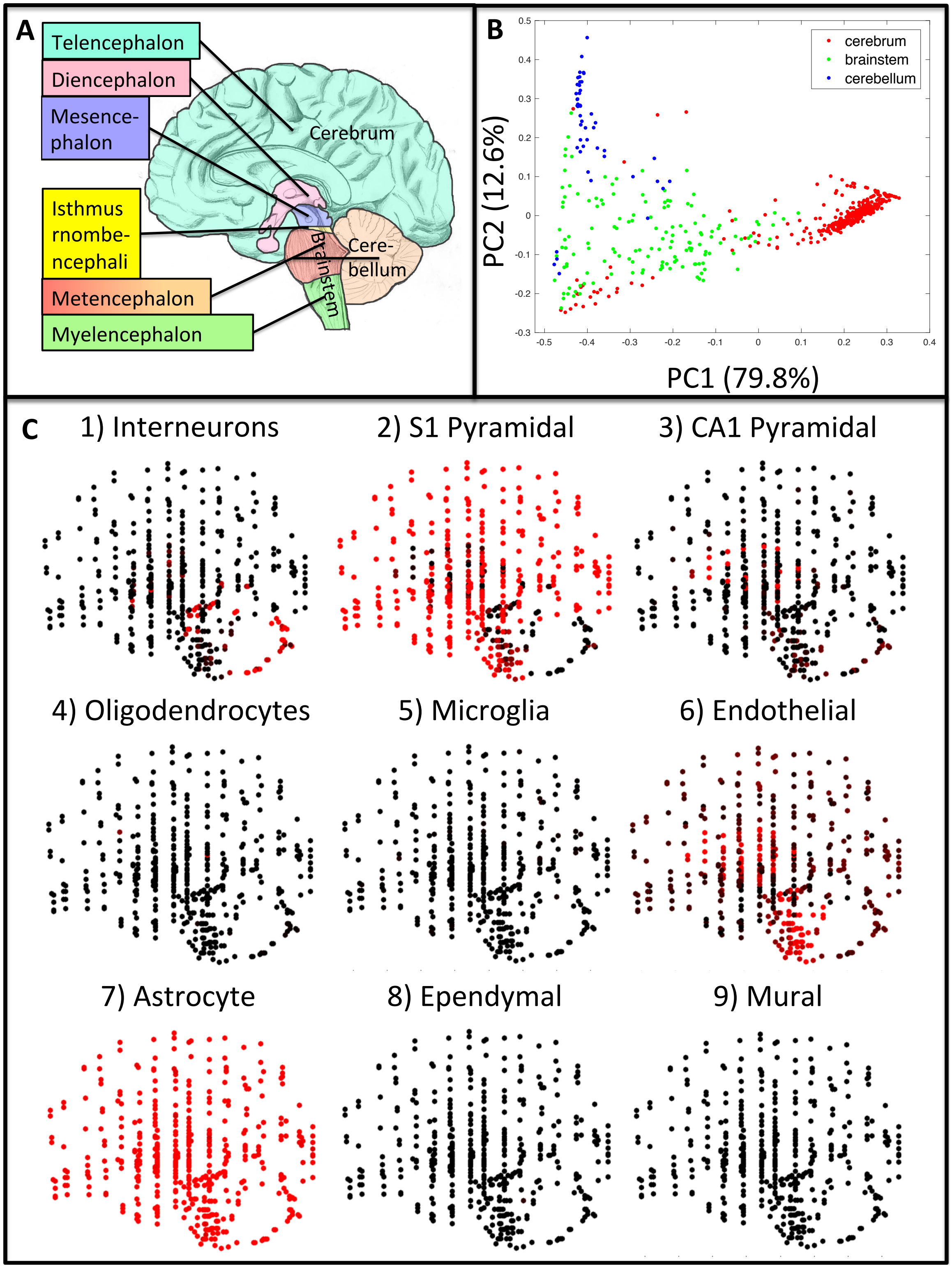

Supplementary Figure 17 Example of spatial distributions of cell type proportions in AHBA donor 14380 using MusNG to deconvolute the RNA expression profile. **A)** A reference map with developmental structures and major brain regions marked. **B)** PCA plot of the cell type proportion matrix such that the 9 cell types are reduced to 2 principal cell types for each of the samples in the brain. The colors indicate the three regions (cerebrum, brainstem, and cerebellum). **C)** The proportion of each cell type plotted from a sagittal view; Red: high cell proportion (1); Black: low cell proportion (0) on a scale 0-1.

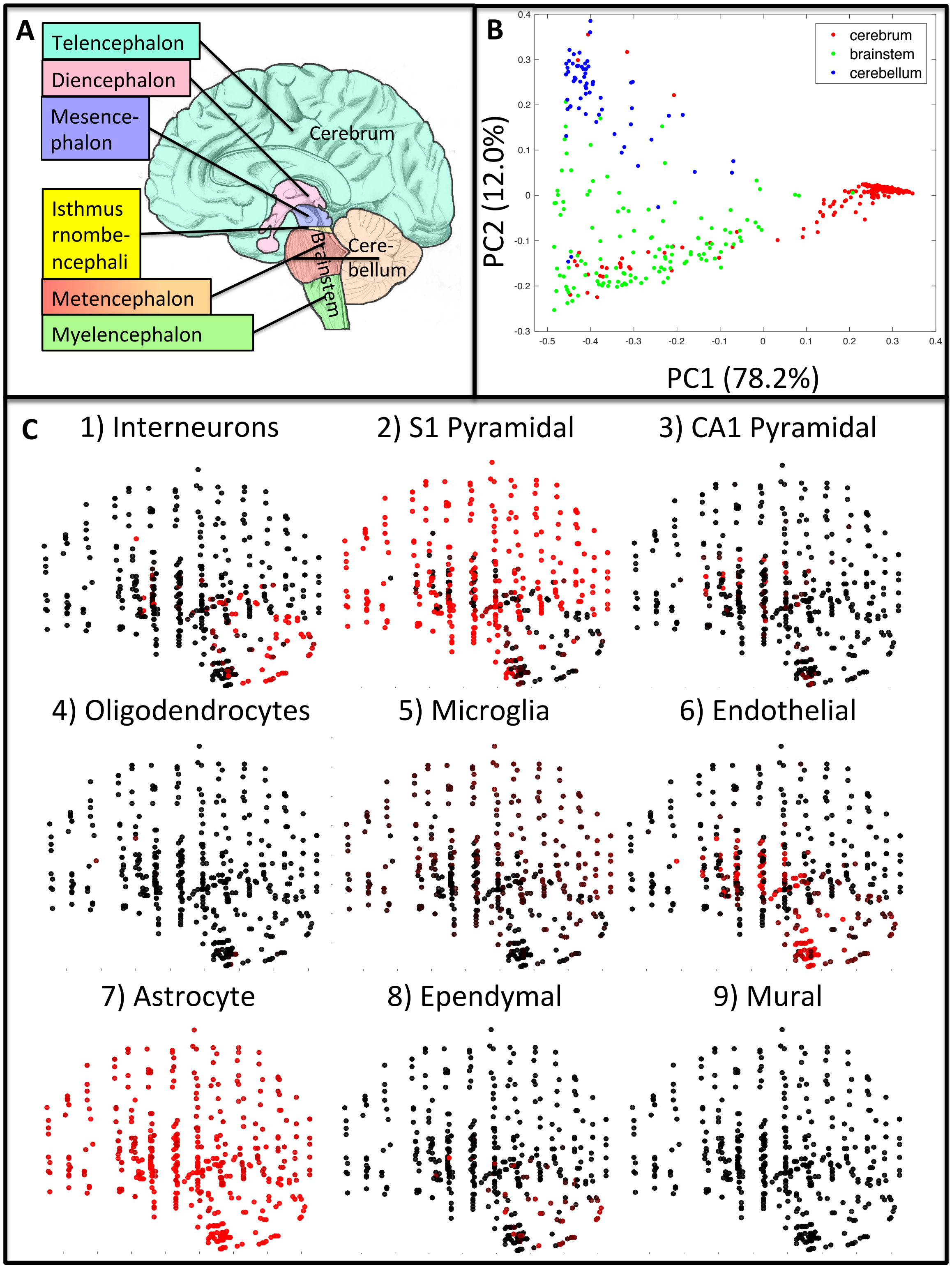

Supplementary Figure 18 Example of spatial distributions of cell type proportions in AHBA donor 15496 using MusNG to deconvolute the RNA expression profile. **A)** A reference map with developmental structures and major brain regions marked. **B)** PCA plot of the cell type proportion matrix such that the 9 cell types are reduced to 2 principal cell types for each of the samples in the brain. The colors indicate the

three regions (cerebrum, brainstem, and cerebellum). **C)** The proportion of each cell type plotted from a sagittal view; Red: high cell proportion (1); Black: low cell proportion (0) on a scale 0-1.

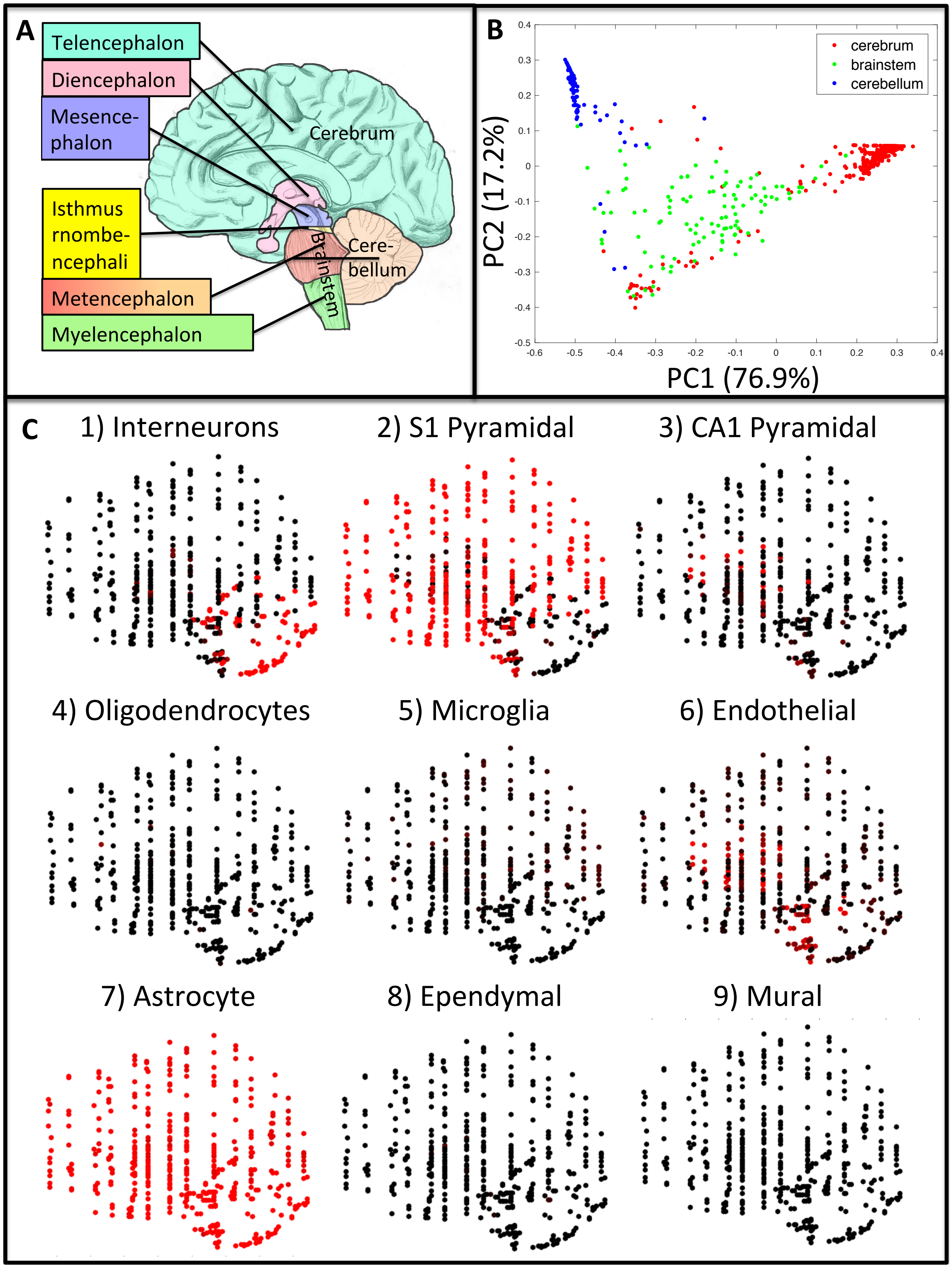

Supplementary Figure 19 Example of spatial distributions of cell type proportions in AHBA donor 15697 using MusNG to deconvolute the RNA expression profile. **A)** A reference map with developmental structures and major brain regions marked. **B)** PCA plot of the cell type proportion matrix such that the 9

cell types are reduced to 2 principal cell types for each of the samples in the brain. The colors indicate the three regions (cerebrum, brainstem, and cerebellum). **C)** The proportion of each cell type plotted from a sagittal view; Red: high cell proportion (1); Black: low cell proportion (0) on a scale 0-1.

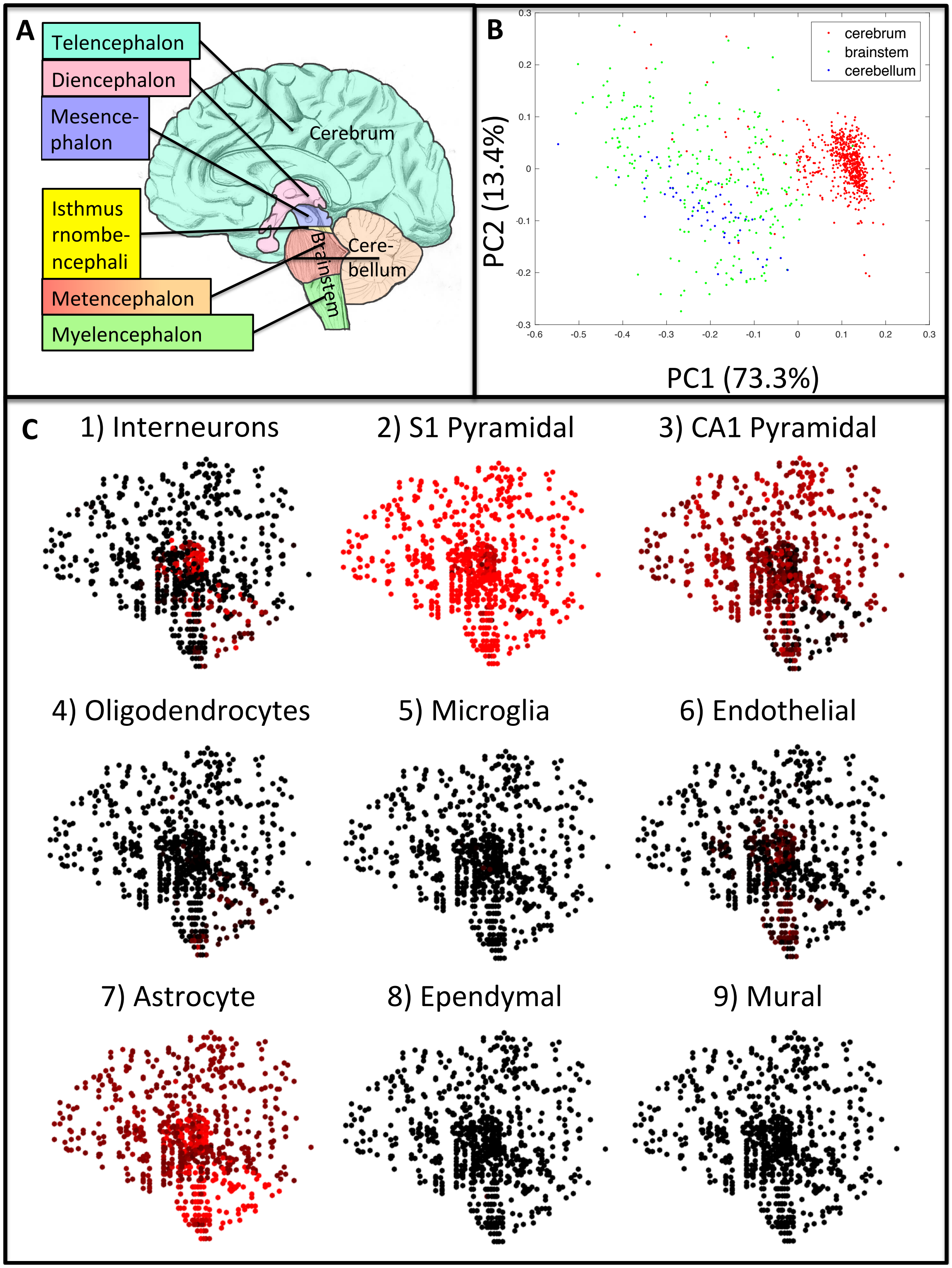

Supplementary Figure 20 Example of spatial distributions of cell type proportions in AHBA donor 9861 using HumN to deconvolute the RNA expression profile. **A)** A reference map with developmental

structures and major brain regions marked. **B)** PCA plot of the cell type proportion matrix such that the 9 cell types are reduced to 2 principal cell types for each of the samples in the brain. The colors indicate the three regions (cerebrum, brainstem, and cerebellum). **C)** The proportion of each cell type plotted from a sagittal view; Red: high cell proportion (1); Black: low cell proportion (0) on a scale 0-1.

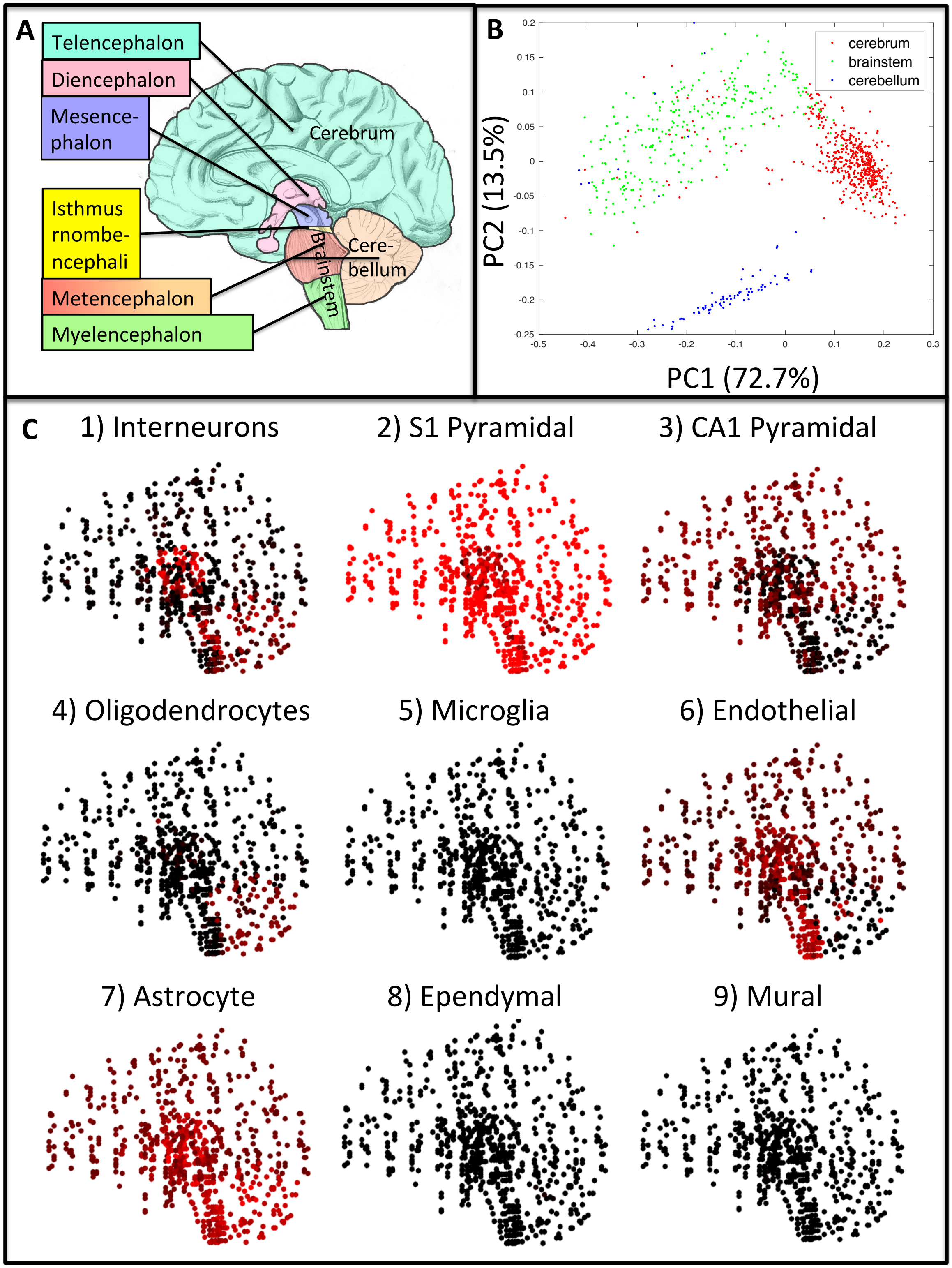

Supplementary Figure 21 Example of spatial distributions of cell type proportions in AHBA donor 10021 using HumN to deconvolute the RNA expression profile. **A)** A reference map with developmental structures and major brain regions marked. **B)** PCA plot of the cell type proportion matrix such that the 9 cell types are reduced to 2 principal cell types for each of the samples in the brain. The colors indicate the three regions (cerebrum, brainstem, and cerebellum). **C)** The proportion of each cell type plotted from a sagittal view; Red: high cell proportion (1); Black: low cell proportion (0) on a scale 0-1.

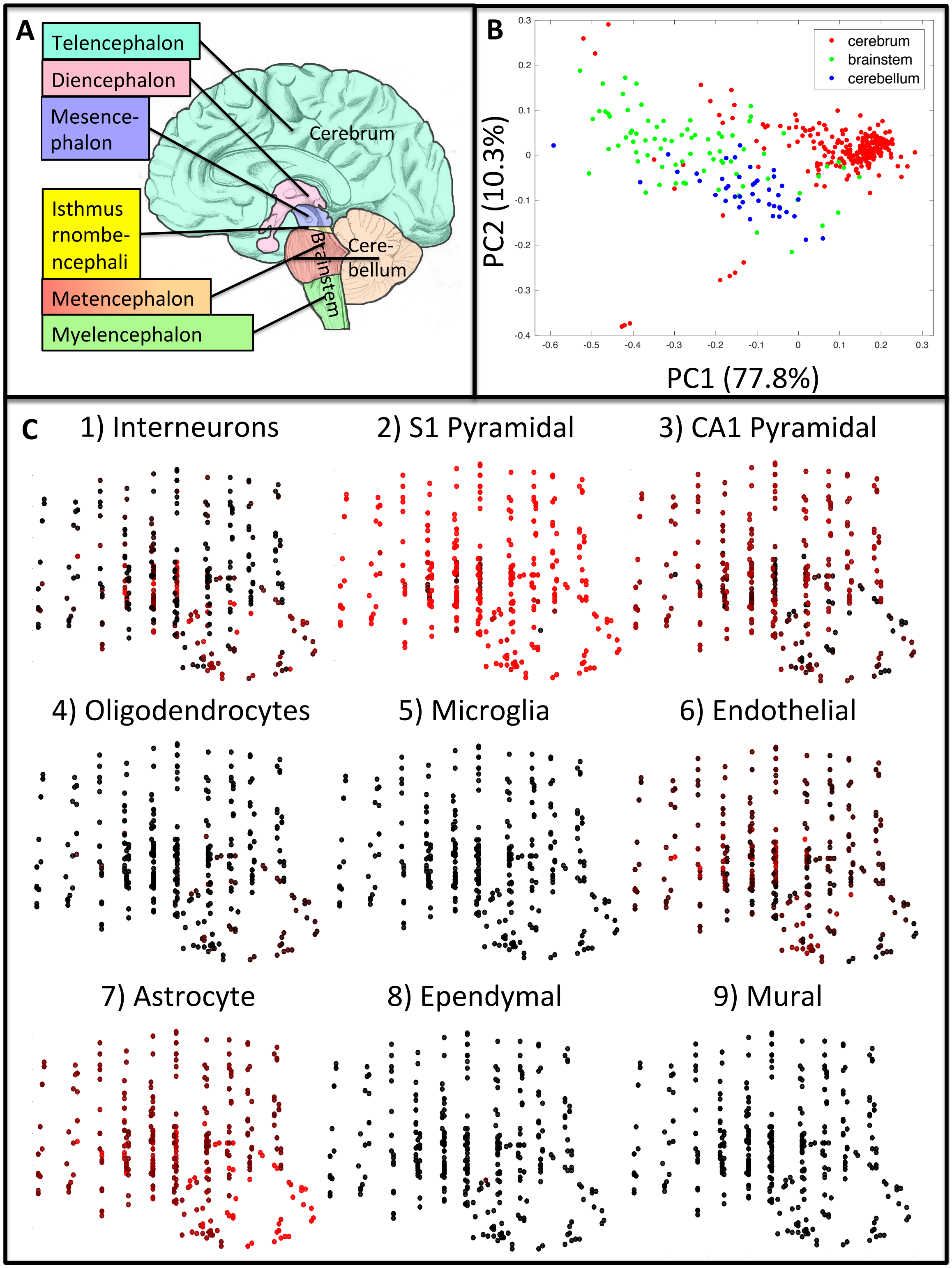

Supplementary Figure 22 Example of spatial distributions of cell type proportions in AHBA donor 12876 using HumN to deconvolute the RNA expression profile. **A)** A reference map with developmental structures and major brain regions marked. **B)** PCA plot of the cell type proportion matrix such that the 9 cell types are reduced to 2 principal cell types for each of the samples in the brain. The colors indicate the

three regions (cerebrum, brainstem, and cerebellum). **C)** The proportion of each cell type plotted from a sagittal view; Red: high cell proportion (1); Black: low cell proportion (0) on a scale 0-1.

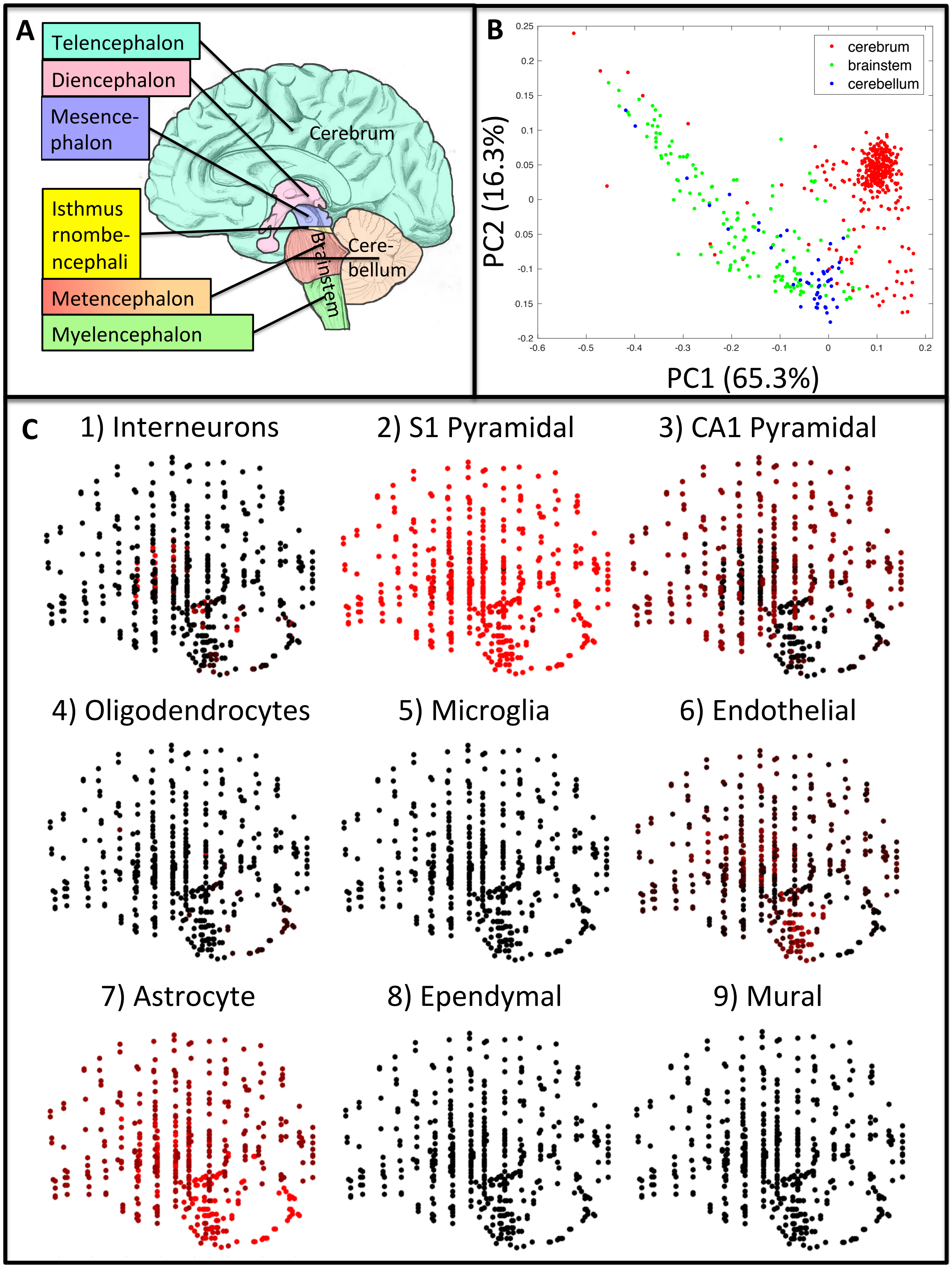

Supplementary Figure 23 Example of spatial distributions of cell type proportions in AHBA donor 14380 using HumN to deconvolute the RNA expression profile. **A)** A reference map with developmental structures and major brain regions marked. **B)** PCA plot of the cell type proportion matrix such that the 9

cell types are reduced to 2 principal cell types for each of the samples in the brain. The colors indicate the three regions (cerebrum, brainstem, and cerebellum). **C)** The proportion of each cell type plotted from a sagittal view; Red: high cell proportion (1); Black: low cell proportion (0) on a scale 0-1.

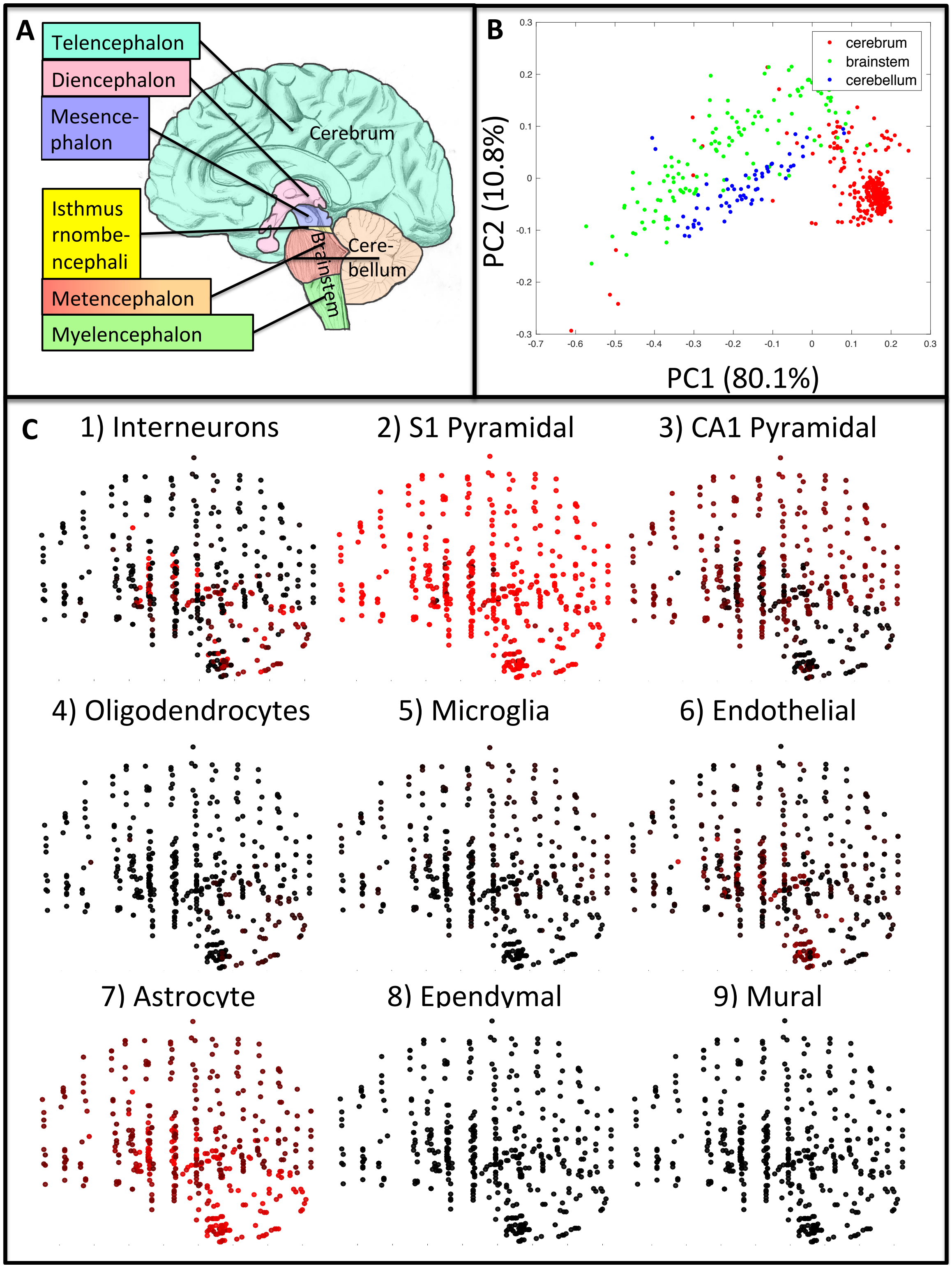

Supplementary Figure 24 Example of spatial distributions of cell type proportions in AHBA donor 15496 using HumN to deconvolute the RNA expression profile. **A)** A reference map with developmental

structures and major brain regions marked. **B)** PCA plot of the cell type proportion matrix such that the 9 cell types are reduced to 2 principal cell types for each of the samples in the brain. The colors indicate the three regions (cerebrum, brainstem, and cerebellum). **C)** The proportion of each cell type plotted from a sagittal view; Red: high cell proportion (1); Black: low cell proportion (0) on a scale 0-1.

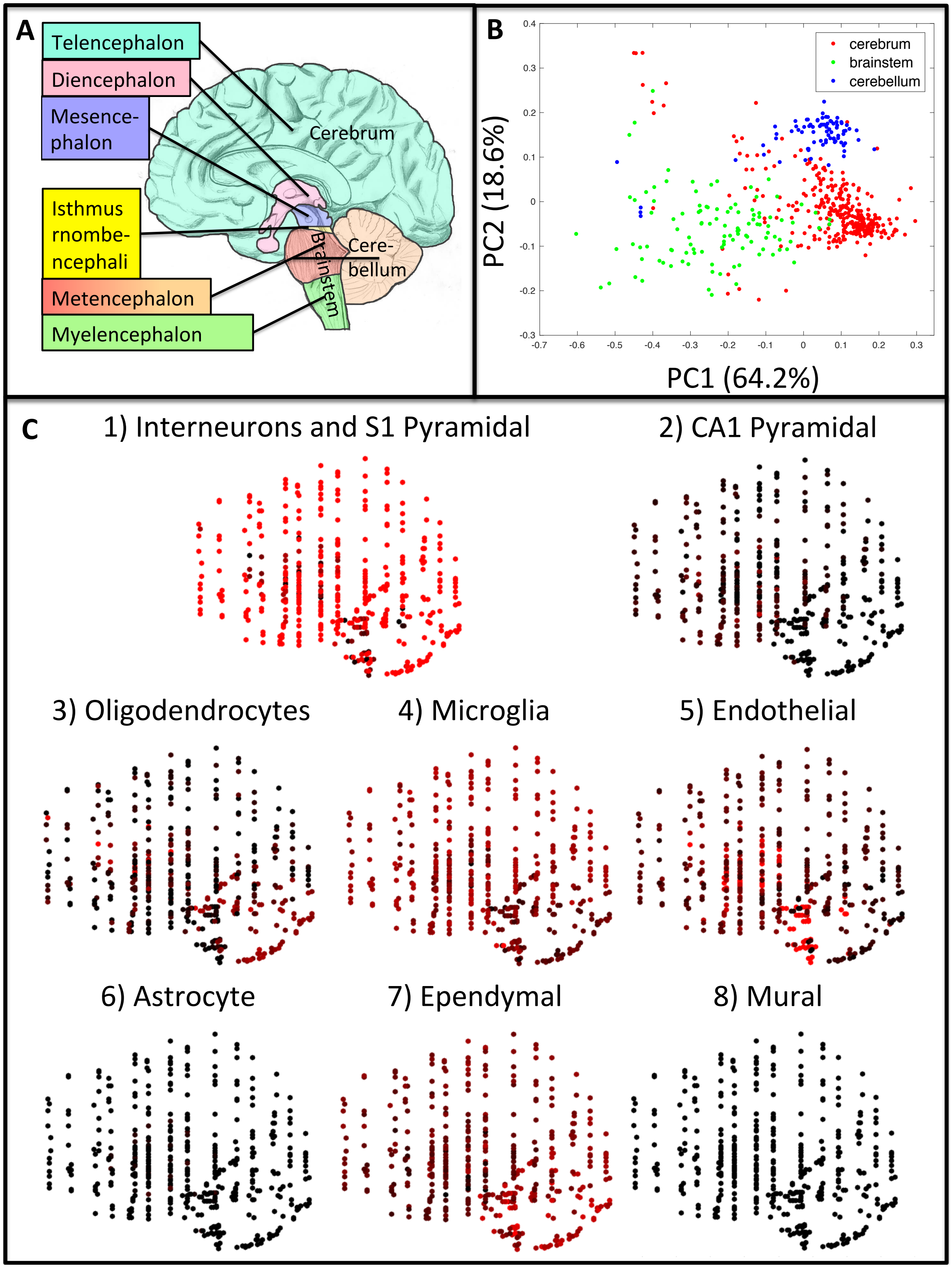

Supplementary Figure 25 Example of spatial distributions of cell type proportions in AHBA donor 15697 using HumN to deconvolute the RNA expression profile. **A)** A reference map with developmental structures and major brain regions marked. **B)** PCA plot of the cell type proportion matrix such that the 9 cell types are reduced to 2 principal cell types for each of the samples in the brain. The colors indicate the three regions (cerebrum, brainstem, and cerebellum). **C)** The proportion of each cell type plotted from a sagittal view; Red: high cell proportion (1); Black: low cell proportion (0) on a scale 0-1.

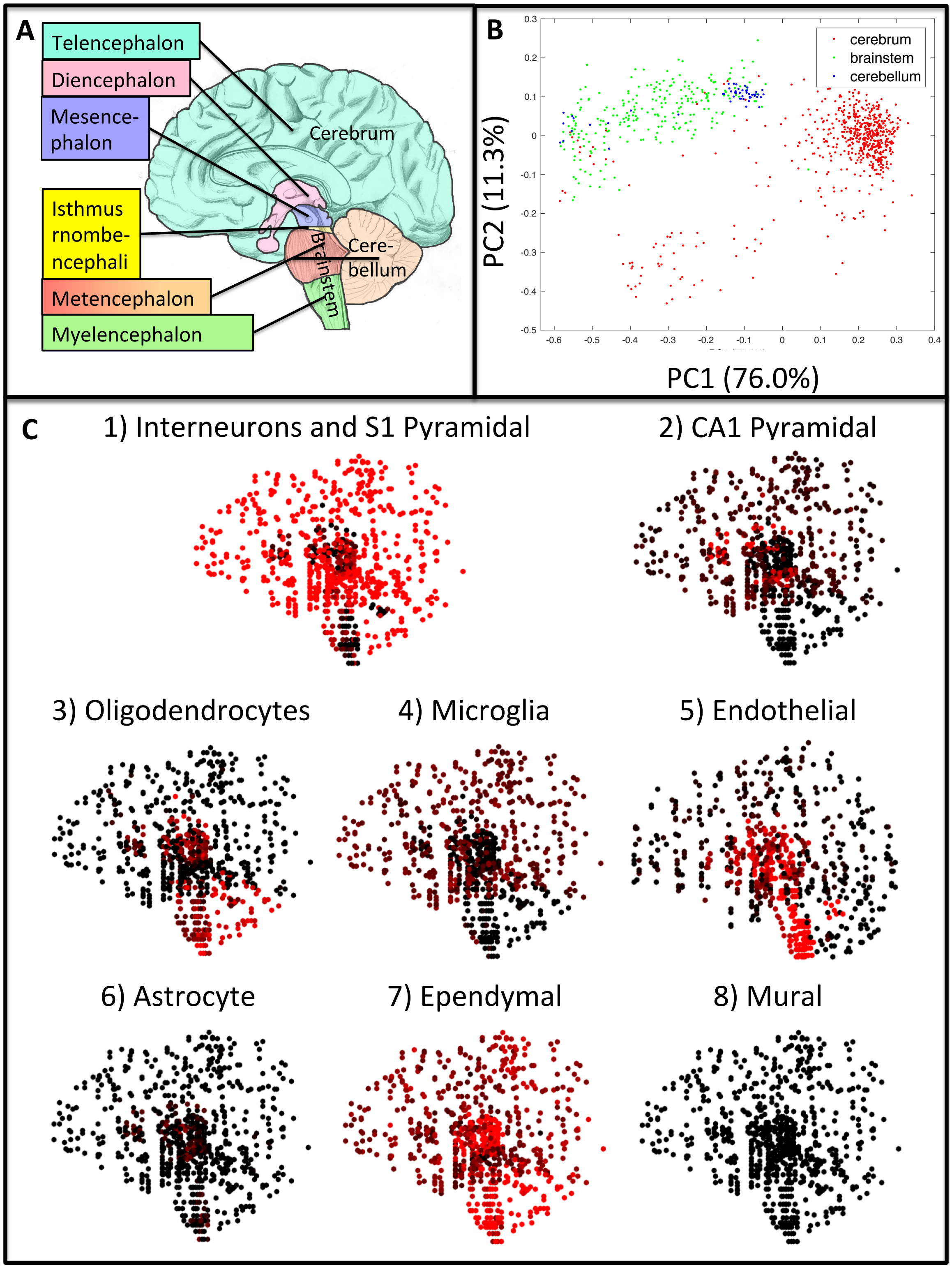

Supplementary Figure 26 Example of spatial distributions of cell type proportions in AHBA donor 9861 using HumNG to deconvolute the RNA expression profile. **A)** A reference map with developmental structures and major brain regions marked. **B)** PCA plot of the cell type proportion matrix such that the 9 cell types are reduced to 2 principal cell types for each of the samples in the brain. The colors indicate the

three regions (cerebrum, brainstem, and cerebellum). **C)** The proportion of each cell type plotted from a sagittal view; Red: high cell proportion (1); Black: low cell proportion (0) on a scale 0-1.

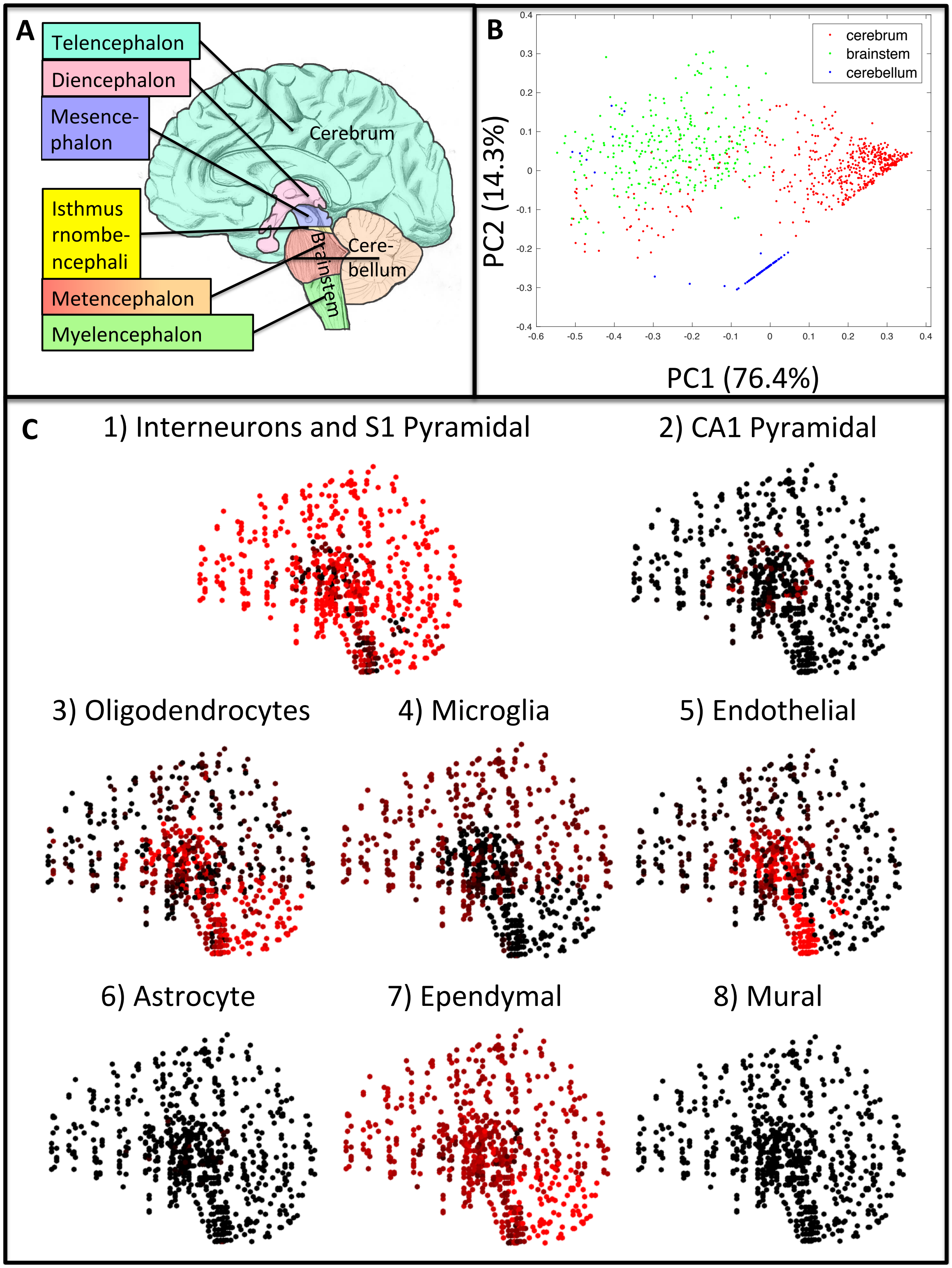

Supplementary Figure 27 Example of spatial distributions of cell type proportions in AHBA donor 10021 using HumNG to deconvolute the RNA expression profile. **A)** A reference map with developmental structures and major brain regions marked. **B)** PCA plot of the cell type proportion matrix such that the 9

cell types are reduced to 2 principal cell types for each of the samples in the brain. The colors indicate the three regions (cerebrum, brainstem, and cerebellum). **C)** The proportion of each cell type plotted from a sagittal view; Red: high cell proportion (1); Black: low cell proportion (0) on a scale 0-1.

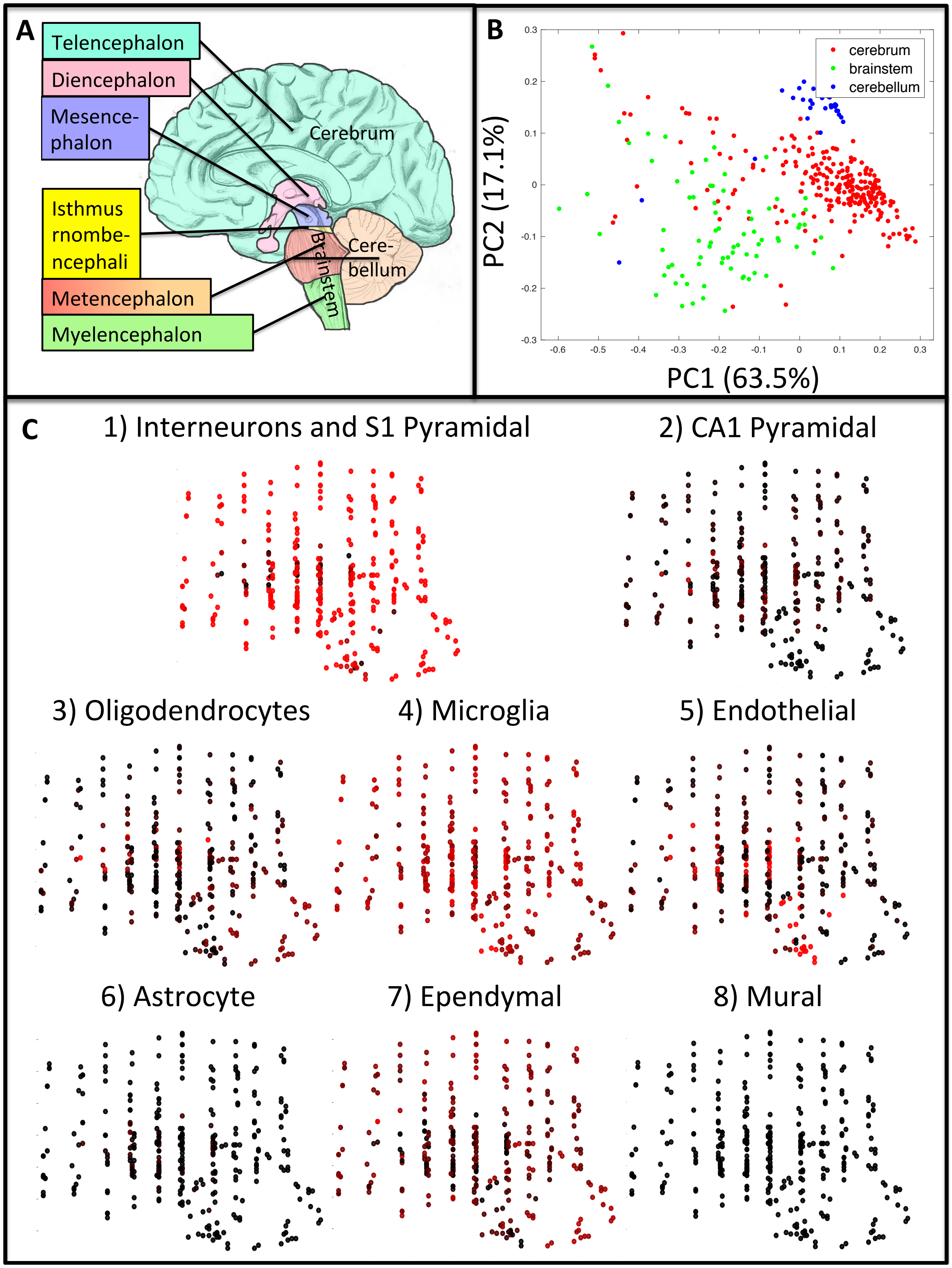

Supplementary Figure 28 Example of spatial distributions of cell type proportions in AHBA donor 12876 using HumNG to deconvolute the RNA expression profile. **A)** A reference map with developmental

structures and major brain regions marked. **B)** PCA plot of the cell type proportion matrix such that the 9 cell types are reduced to 2 principal cell types for each of the samples in the brain. The colors indicate the three regions (cerebrum, brainstem, and cerebellum). **C)** The proportion of each cell type plotted from a sagittal view; Red: high cell proportion (1); Black: low cell proportion (0) on a scale 0-1.

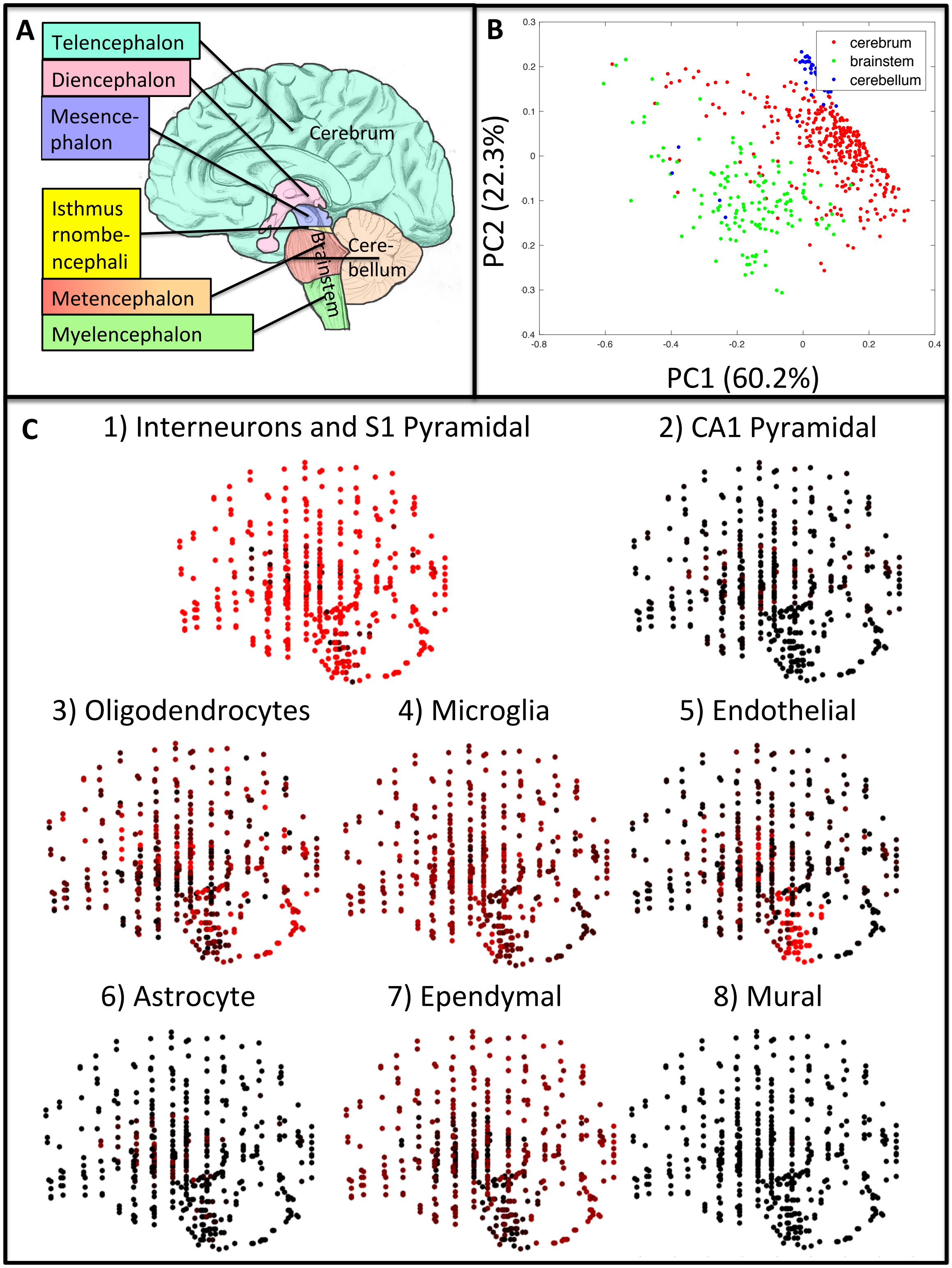

Supplementary Figure 29 Example of spatial distributions of cell type proportions in AHBA donor 14380 using HumNG to deconvolute the RNA expression profile. **A)** A reference map with developmental structures and major brain regions marked. **B)** PCA plot of the cell type proportion matrix such that the 9 cell types are reduced to 2 principal cell types for each of the samples in the brain. The colors indicate the three regions (cerebrum, brainstem, and cerebellum). **C)** The proportion of each cell type plotted from a sagittal view; Red: high cell proportion (1); Black: low cell proportion (0) on a scale 0-1.

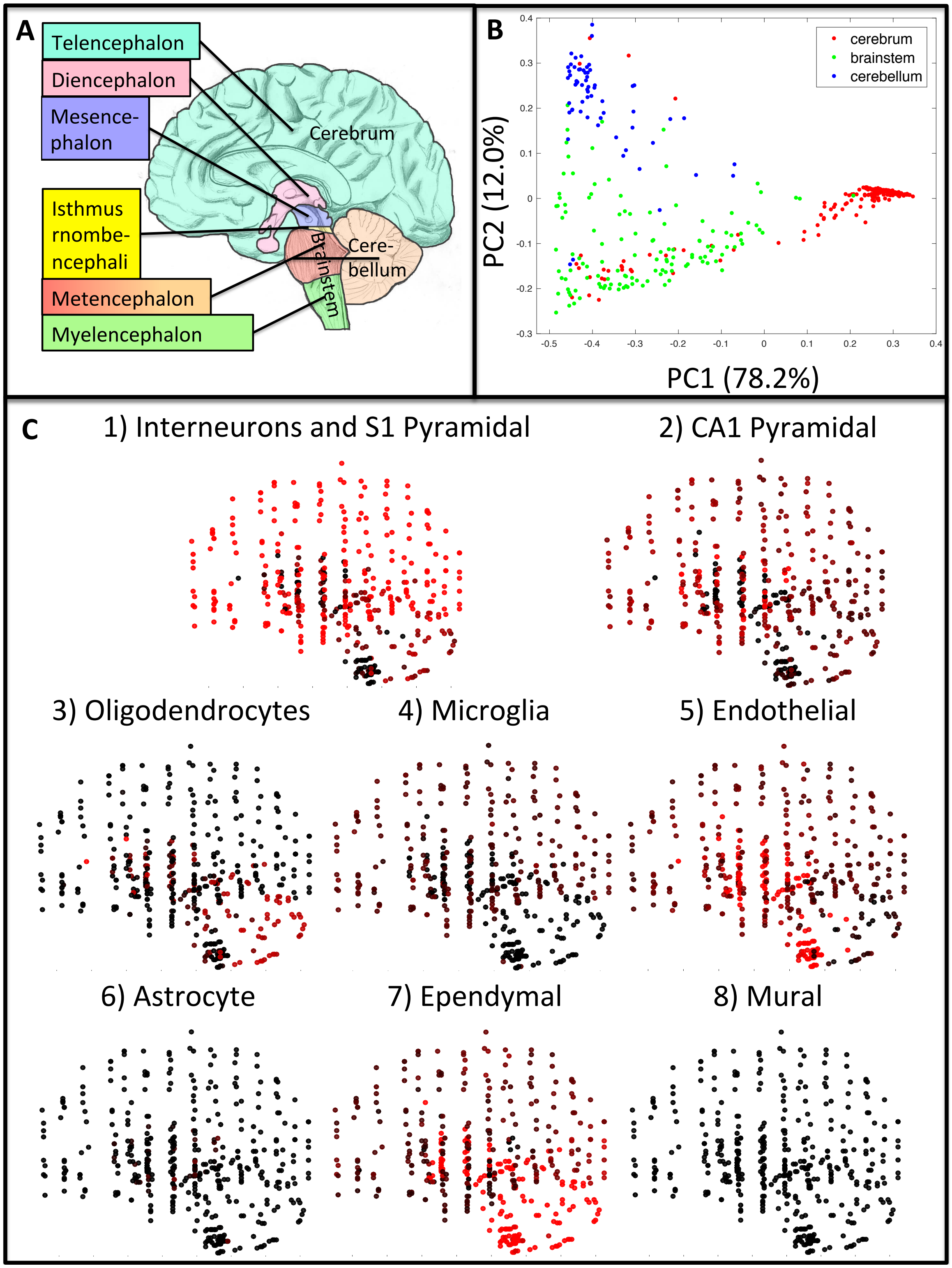

Supplementary Figure 30 Example of spatial distributions of cell type proportions in AHBA donor 15496 using HumNG to deconvolute the RNA expression profile. **A)** A reference map with developmental structures and major brain regions marked. **B)** PCA plot of the cell type proportion matrix such that the 9 cell types are reduced to 2 principal cell types for each of the samples in the brain. The colors indicate the

three regions (cerebrum, brainstem, and cerebellum). **C)** The proportion of each cell type plotted from a sagittal view; Red: high cell proportion (1); Black: low cell proportion (0) on a scale 0-1.

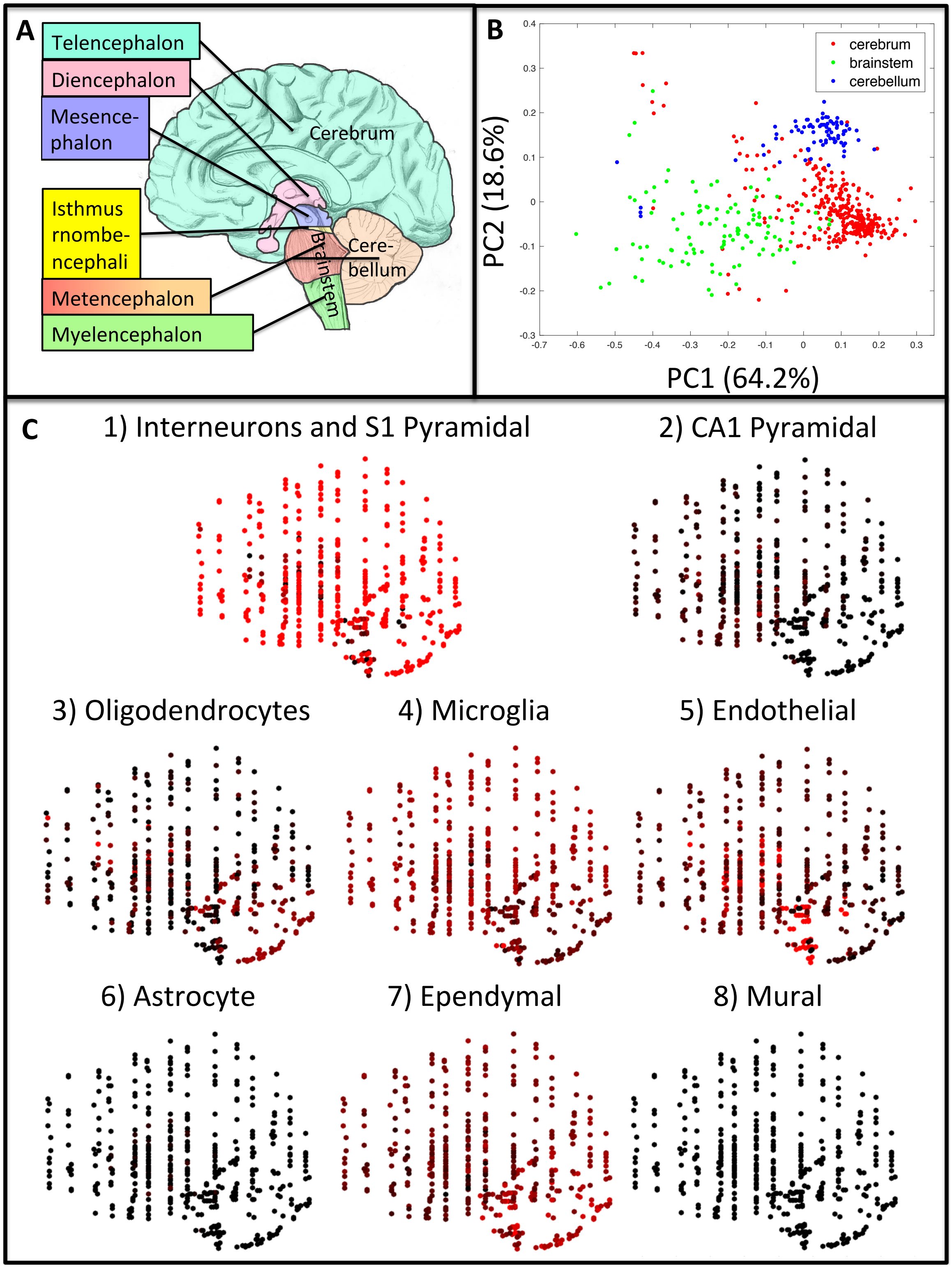

Supplementary Figure 31 Example of spatial distributions of cell type proportions in AHBA donor 15697 using HumNG to deconvolute the RNA expression profile. **A)** A reference map with developmental structures and major brain regions marked. **B)** PCA plot of the cell type proportion matrix such that the 9

cell types are reduced to 2 principal cell types for each of the samples in the brain. The colors indicate the three regions (cerebrum, brainstem, and cerebellum). **C)** The proportion of each cell type plotted from a sagittal view; Red: high cell proportion (1); Black: low cell proportion (0) on a scale 0-1.

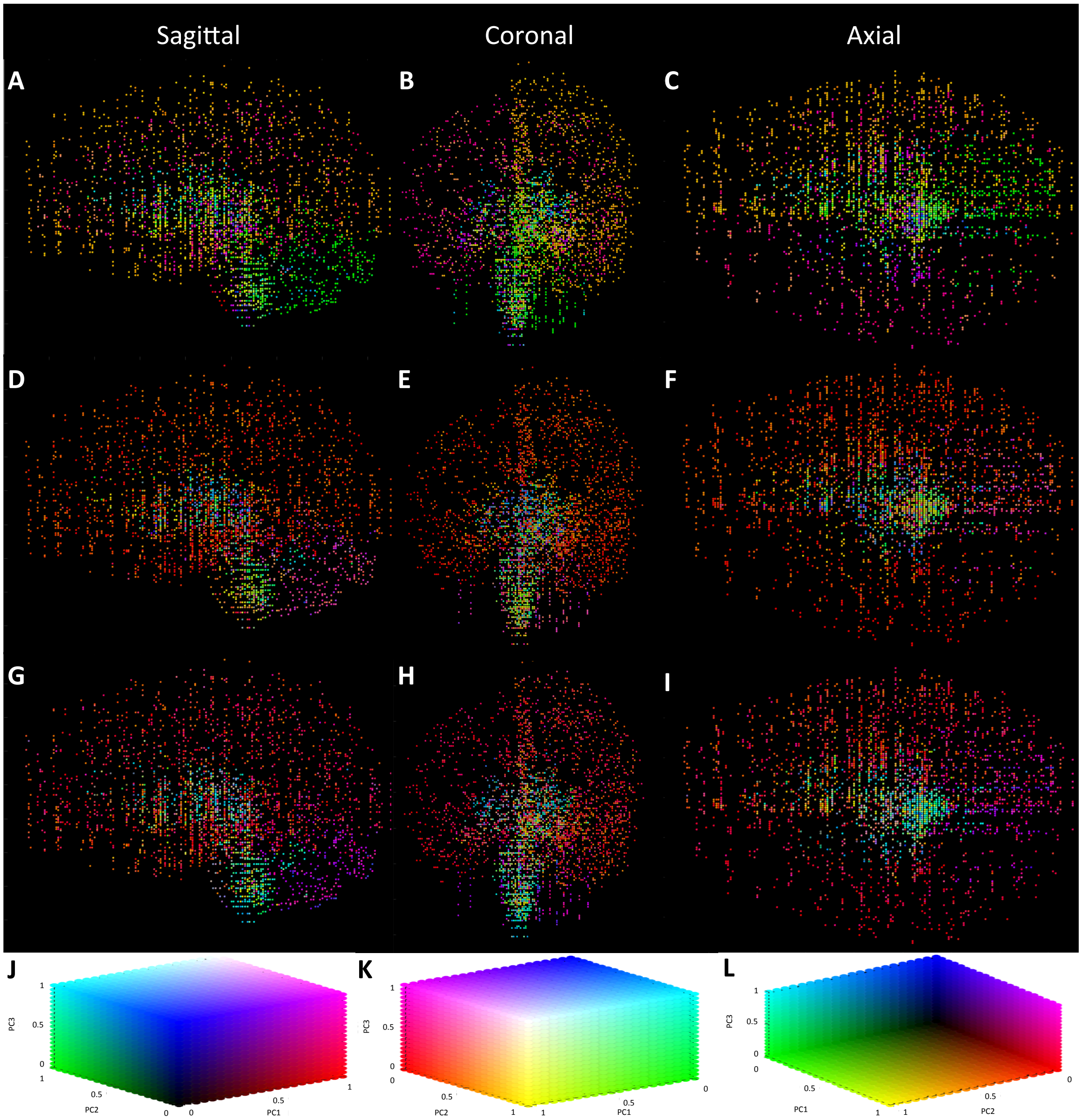

Supplementary Figure 32 The 3D spatial mapping of major cell types from Allen Human Brain Atlas (AHBA) donors. Six samples are stacked together. The colors represent the first three principal cell types derived from principal component analysis (PCA) on the cell-type proportion matrix. **A, B, C)** MusNG deconvolution; **D, E, F)** HumN deconvolution; and **G, H, I)** HumNG deconvolution.

	D9861	D10021	D12876	D14380	D15496	D15697
MusNG-HumN	8.5415×10^{-15}	2.1110×10^{-14}	9.4471×10^{-12}	9.2133×10^{-12}	3.2302×10^{-08}	1.5885×10^{-09}
MusNG-	2.5544×10^{-04}	5.1148×10^{-10}	8.7296×10^{-06}	5.9451×10^{-06}	1.5071×10^{-05}	1.9738×10^{-06}

HumNG						
HumN-HumNG	3.0739×10^{-05}	7.8195×10^{-06}	7.1236×10^{-08}	6.6163×10^{-06}	3.3409×10^{-06}	1.2331×10^{-08}

Supplementary Table I Each row is the combination scRNA-Seq datasets being compared. Each column is a brain donor. The cells contain p-values from an anova of cell type correlation values grouped by 1) same cell type-between datasets versus 2) different cell type-between datasets (within dataset same cell type correlations removed because they have a correlation of 1). See Consistency of Cell Type Proportions Between Datasets in the results section for a detailed description of how these p-values were obtained.

	Accuracy - overall	Sens-cerebrum	Sens - brainstem	Sens - cerebellum	Spec – cerebrum	Spec – brainstem	Spec - cerebellumq	Fscore – cerebrum	Fscore – brainstem	Fscore - cerebellum
MusNG-d9861	0.7030	0.7994	0.4080	0.9245	0.8581	0.8132	0.8791	0.8567	0.4232	0.4667
MusNG-d10021	0.8813	0.8409	0.9539	0.8916	0.9671	0.8478	0.9988	0.9024	0.8354	0.9367
MusNG-d12876	0.8182	0.8642	0.6026	0.9524	0.9500	0.8877	0.9128	0.9150	0.5987	0.7273
MusNG-d14380	0.8696	0.9021	0.8056	0.8333	0.9479	0.9039	0.9543	0.9339	0.7811	0.7273
MusNG-d15496	0.8830	0.8936	0.8492	0.9032	0.9681	0.9070	0.9583	0.9333	0.8075	0.8296
MusNG-d15697	0.8902	0.8810	0.8727	0.9500	0.9526	0.9028	0.9810	0.9226	0.7869	0.9268
HumN-d9861	0.8171	0.9565	0.5720	0.2830	009637	0.9167	0.8835	0.9693	0.6341	0.1744
HumN-d10021	0..8992	0.9451	0.8191	0.8795	0.8575	0.9378	1.0000	0.9249	0.8385	0.9359
HumN-d12876	0.8072	0.8765	0.5513	0.8810	0.9333	0.9579	0.8442	0.9181	0.6466	0.5736
HumN-d14380	0.7675	0.9169	0.3750	0.8958	0.9844	0.9662	0.7775	0.9522	0.5118	0.4343
HumN-d15496	0.7617	0.9468	0.5238	0.4032	0.9628	0.8750	0.8480	0.9604	0.5617	0.3356
HumN-d15697	0.8663	0.9357	0.7182	0.8000	0.8947	0.9156	0.9667	0.9357	0.7117	0.8101
HumNG-d9861	0.7600	0.8725	0.4640	0.7925	0.9967	0.9066	0.8197	0.9311	0.5383	0.3281
HumNG-d10021	0.7794	0.7727	0.7553	0.9036	0.9973	0.8985	0.8346	0.8709	0.7648	0.5137
HumNG-d12876	0.6915	0.6091	0.8077	0.9524	0.9583	0.8807	0.7726	0.7475	0.7200	0.5161
HumNG-d14380	0.6786	0.5668	0.8611	0.9167	0.9062	0.9117	0.7547	0.6996	0.8212	0.4190
HumNG-d15496	0.9234	0.9433	0.8651	0.9516	1.0000	0.9506	0.9534	0.9708	0.8651	0.8429
HumNG-d15697	0.8283	0.7846	0.8727	0.9375	0.9368	0.9182	0.9002	0.8607	0.8067	0.7614

Supplementary Table II Each row is a combination of scRNA-Seq dataset and brain donor each corresponds to a spatial distribution figure (e.g. Figure 5B, Supplementary Figures 14-30B). The overall accuracy for k-means clusters as well as the cluster-wise sensitivity, cluster-wise specificity, and cluster-wise F-score are contained in each column.

	Inter-neuron	S1 Pyramidal	CA1 Pyramidal	Oligo-dendrocyte	Microglia	Endothelial	Astrocyte	Ependymal	Mural
MusNG-PC1	0.0000	0.8503	0.0043	0.0000	0.0220	0.0000	0.1234	0.0000	0.0000
MusNG-PC2	0.1661	0.0000	0.0000	0.0155	0.0089	0.2815	0.5083	0.0196	0.0000
MusNG-PC3	0.0484	0.1032	0.6581	0.0070	0.0062	0.1666	0.0000	0.0104	0.0000
HumN-PC1	0.0000	0.6279	0.2020	0.0041	0.0064	0.0000	0.1596	0.0000	0.0000
HumN-PC2	0.0000	0.3645	0.0000	0.0075	0.0000	0.3499	0.2780	0.0000	0.0000
HumN-	0.4871	0.0683	0.0180	0.0609	0.0000	0.0145	0.3507	0.0005	0.000

PC3									0
HumNG-PC1	0.6156*	0.6156*	0.0680	0.0000	0.1918	0.0000	0.0000	0.1245	0.0000
HumNG-PC2	0.0075*	0.0075*	0.0747	0.0301	0.0584	0.6839	0.0314	0.1136	0.0003
HumNG-PC3	0.0899*	0.0899*	0.0000	0.4358	0.0000	0.0180	0.0060	0.4503	0.0000

Supplementary Table III Cell type makeup of each of the 3 principal cell types used to generate Figure 7. * In the HumNG dataset there are only 8 cell types because the interneuron and S1 pyramidal were combined. The percentages in those cells are for the combined cell type.

Bibliography

1 Zeisel, A. *et al.* Brain structure. Cell types in the mouse cortex and hippocampus revealed by single-cell RNA-seq. *Science* **347**, 1138-1142, doi:10.1126/science.aaa1934 (2015).

2 Ding, C. & Peng, H. Minimum redundancy feature selection from microarray gene expression data. *J Bioinform Comput Biol* **3**, 185-205 (2005).

3 Dinai, Y., Wolf, L. & Assaf, Y. Combined neuroimaging and gene expression analysis of the genetic basis of brain plasticity indicates across species homology. *Hum Brain Mapp* **35**, 5888-5902, doi:10.1002/hbm.22592 (2014).

4 Johnson, T., Abrams, Z., Zhang, Y. & Huang, K. Mapping Neuronal Cell Types Using Integrative Multi-Species Modeling of Human and Mouse Single Cell Rna Sequencing. *Pac Symp Biocomput* **22**, 599-610 (2016).

5 Venet, D., Pecasse, F., Maenhaut, C. & Bersini, H. Separation of samples into their constituents using gene expression data. *Bioinformatics* **17 Suppl 1**, S279-287 (2001).

6 Liebner, D. A., Huang, K. & Parvin, J. D. MMAD: microarray microdissection with analysis of differences is a computational tool for deconvoluting cell type-specific contributions from tissue samples. *Bioinformatics* **30**, 682-689, doi:10.1093/bioinformatics/btt566 (2014).

7 Li, C. & Hung Wong, W. Model-based analysis of oligonucleotide arrays: model validation, design issues and standard error application. *Genome Biol* **2**, RESEARCH0032 (2001).

8 Jolliffe, I. T. A note on the Use of Principal Components in Regression. *Journal of the Royal Statistical Society, Series C* **31**, 300-303, doi:10.2307/2348005 (1982).

9 Berry M W, B. M., Langville AN, Pauca VP, Plemmons RJ. Algorithms and applications for approximate nonnegative matrix factorization. *Comput Stat Data Anal* **52**, 155-173 (2007).

10 Lee D, S. H. Algorithms for non-negative matrix factorization. *Adv Neural Inf Process Syst* (2001).
